# Unveiling the DHX15–G-patch interplay in retroviral RNA packaging

**DOI:** 10.1073/pnas.2407990121

**Published:** 2024-09-25

**Authors:** Alžběta Dostálková, Ivana Křížová, Petra Junková, Jana Racková, Marina Kapisheva, Radim Novotný, Matěj Danda, Karolína Zvonařová, Larisa Šinkovec, Kateřina Večerková, Lucie Bednářová, Tomáš Ruml, Michaela Rumlová

**Affiliations:** ^a^Department of Biotechnology, University of Chemistry and Technology, 166 28 Prague, Czech Republic; ^b^Institute of Organic Chemistry and Biochemistry Research Centre & Gilead Sciences, Czech Academy of Sciences, 166 10 Prague, Czech Republic; ^c^Department of Biochemistry and Microbiology, University of Chemistry and Technology 166 28, Prague, Czech Republic; ^d^Department of Informatics and Chemistry, University of Chemistry and Technology, 166 28 Prague, Czech Republic; ^e^Institute of Molecular Genetics, Czech Academy of Sciences, 142 20 Prague, Czech Republic

**Keywords:** DEAH-box RNA helicase, DHX15, G-patch, retrovirus, gRNA packaging

## Abstract

We explored how a simple retrovirus, Mason-Pfizer monkey virus (M-PMV) to facilitate its replication process, utilizes DHX15, a cellular RNA helicase, typically engaged in RNA processing. Through advanced genetic engineering techniques, we showed that M-PMV recruits DHX15 by mimicking cellular mechanisms, relocating it from the nucleus to the cytoplasm to aid in viral assembly. This interaction is essential for the correct packaging of the viral genome and critical for its infectivity. Our findings offer unique insights into the mechanisms of viral manipulation of host cellular processes, highlighting a sophisticated strategy that viruses employ to leverage cellular machinery for their replication. This study adds valuable knowledge to the understanding of viral–host interactions but also suggests a common evolutionary history between cellular processes and viral mechanisms. This finding opens a unique perspective on the export mechanism of intron-retaining mRNAs in the packaging of viral genetic information and potentially develop ways to stop it.

## Significance

Our findings define a unique role for the cellular DEAH-box RNA helicase DHX15. We found that a viral G-patch motif functions as a cofactor, recruiting DHX15 into Mason-Pfizer monkey virus (M-PMV) particles. DHX15 plays a pivotal role in M-PMV replication by facilitating the packaging of genomic RNA (gRNA) into virions. Notably, the interaction of DHX15 with the constitutive transport element region of gRNA highlights its importance in gRNA packaging. These findings advance our understanding of the interplay between the retroviral G-patch and DHX15, providing valuable insights into viral replication. Moreover, they shed light onto a possible unique function for a cellular DEAH-box helicase and offer insight into potential antiviral strategies.

Cellular RNA helicases are fundamental molecular machines that participate in all aspects of nucleic acid metabolism (1–3). They use ATP to remodel nucleic acids and nucleic acid–protein complexes (ribonucleoprotein particles or RNPs) (3–5). Within the broad spectrum of RNA helicases, two from the DExD/H group, DEAD-box and DEAH-box, are particularly intriguing due to their distinct mechanisms of RNA remodeling and extensive roles in diverse cellular processes (1, 5–7). They share a core helicase architecture formed by the tandem RecA-like domains RecA1 and RecA2, which are responsible for ATP binding and hydrolysis and RNA binding and unwinding (3). Yet they differ in their modes of RNA remodeling. DExD-box helicases bind a double-strand RNA substrate and unwind short duplex RNA, while DEAH/RHA-box helicases translocate in a 3′ to 5′ direction along single-strand RNA (ssRNA) to unwind an RNA duplex (8–11). DEAH/RHA-box helicases can disrupt long stretches of base pairing. They also execute diverse functions, including RNA strand annealing, to nucleate RNP assembly by RNA clamping or to displace RNA-bound proteins from their substrate (1, 12–17).

**Fig. 1.**
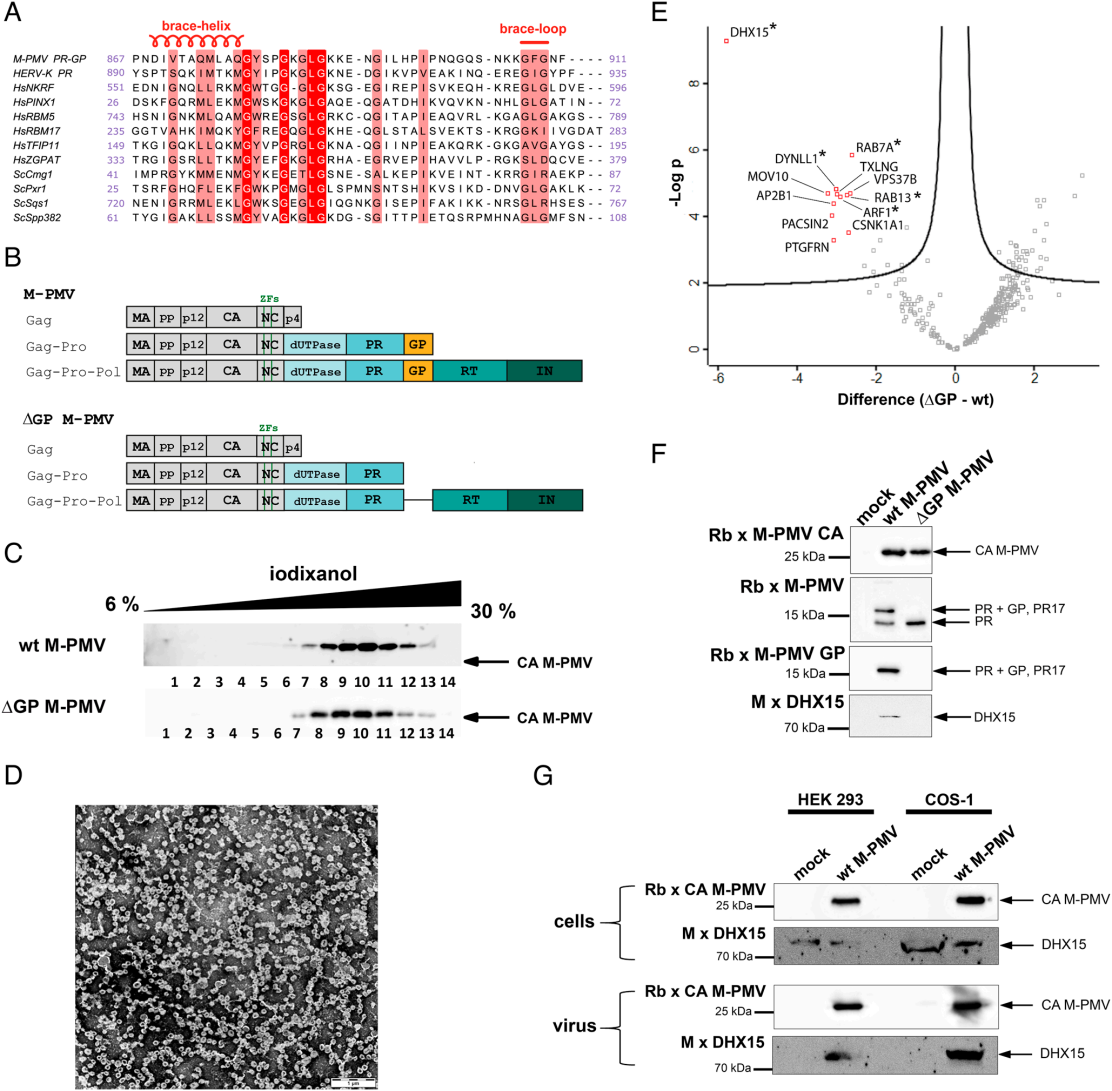
Association of DHX15 with M-PMV. (*A*) Alignment of the conserved amino acids of betaretroviral G-patch proteins (M-PMV, HERV-K), human G-patch proteins (sNKRF, HsPINX1, HsRBM5, HsRBM17, HsTFIP11, HsZGPAT) and yeast G-patch proteins (ScCmg1, ScPxr1, ScSqs1, ScSpp382), all of which interact with DHX15/Prp43. The secondary structural elements, brace helix and brace loop, are indicated. (*B*) Domain organization of wt (*Top* panel) and ΔGP M-PMV (*Bottom* panel). The Gag domains are shown: matrix (MA), phosphoprotein (pp), protein p12, capsid (CA), nucleocapsid (NC) with two zinc finger motifs (ZFs), and protein p4). Pro comprises dUTPase, protease (PR), and the G-patch (GP), and Pol includes reverse transcriptase (RT) and integrase (IN). (*C*) Western blots of individual fractions from the iodixanol gradient used as the final step in wt (*Top* panel) and ΔGP M-PMV (*Bottom* panel) purification. M-PMV was detected with an antibody against CA. (*D*) Transmission electron microscopy (TEM) analysis of negatively stained wt M-PMV particles obtained by combining fractions 8 to 11 from the iodixanol gradient. (*E*) Volcano plot showing the differences in protein abundance in HEK 293 cells infected with ΔGP and wt M-PMV. The difference represents the log2 fold change of protein abundance plotted against −log10 adjusted *P*-value. Each box represents an individual protein. Proteins above the line change with an adjusted *P*-value < 0.01. Protein boxes significantly decreased in ΔGP M-PMV are marked in red (for protein names, see *SI Appendix*, Table S1). Proteins marked with asterisks were identified in all nine samples of wt M-PMV and none of the ΔGP M-PMV samples. (*F*) Western blots of purified wt and ΔGP M-PMV. Rabbit antibodies against CA, PR, and GP were used to identify viral proteins. A mouse antibody against DHX15 was used to identify DHX15 association with purified virions. (*G*) Western blots of production and release of wt M-PMV viral particles produced in HEK 293 and COS-1 cells.

Given their pivotal roles, dysregulation of the activities of these helicases may result in various cellular malfunctions. As many RNA helicases in these subfamilies show low substrate specificity and can transition to an enzymatically active conformation upon nonspecific RNA interaction, cells have developed several levels of regulation to limit unwanted unwinding activity (10, 15). Accessory proteins, or cofactors, that determine RNA helicase specificity play a central role in this process (15). Recent progress has led to improved characterization of one class of such cofactors, the G-patch-containing proteins (Fig. 1*A*), which regulate the function of a subset of DEAH/RHA-box RNA helicases (for review, see ref. 10). G-patch motifs are characterized by a glycine-rich stretch that spans approximately 45 amino acids, with consensus sequence Gx_2_hhx_3_Gax_2_GxGlGx_3_pxux_3_sx_10-16_GhG, where G is glycine, h is a hydrophobic residue, a is aromatic, l is aliphatic, s is small, u is tiny, and x is any residue (18). While the G-patch was initially viewed as a recruitment platform for DEAH helicases, it is now recognized as a helicase cofactor, possessing the ability to activate the ATPase and helicase functions and thereby contribute to RNP remodeling. Of the 15 known human DEAH-box helicases, only DHX15 and DHX16 (and their yeast counterparts, Prp43 and Prp2) are known to be modulated, activated, and sequestered by G-patch proteins (19). This interplay between G-patch proteins and DEAH-box helicases is vital for their multifunctionality, enabling their involvement in numerous distinct cellular processes, including pre-mRNA splicing, ribosome biogenesis, pre-mRNA capping, transcription regulation, telomerase regulation, and innate immunity (7, 10, 15). Recent structural characterization of two G-patch/helicase complexes, NFKB Repressing Factor (NKRF) G-patch with DHX15 and Spp2 G-patch with Prp2 (a DHX16 homolog), has expanded our understanding of how G-patch modulates DEAH-box RNA helicases (20, 21).

RNA helicases play crucial roles during viral infections. They have a wide range of activities, from their function as viral RNA sensors to their assistance in RNA viruses, which have no endogenous helical activity. While most positive-sense RNA viruses possess helicase activity, retroviruses, including HIV-1, depend entirely on cellular helicases to replicate (22, 23). Several DEAD-box or DEAH-box helicases are involved in regulating various phases of the HIV-1 replication cycle (24, 25). Interestingly, Mason-Pfizer monkey virus (M-PMV), a member of the betaretroviral family, is unique among viruses by encoding a G-patch motif (18). The unspliced genomic RNA (gRNA) of M-PMV is exported from the nucleus to the cytosol via the CTE/NXF1 pathway. In the cytosol, this gRNA is either packaged into new viral particles or used as a template for synthesizing three polyprotein precursors: Gag, Gag-Pro, and Gag-Pro-Pol. These polyproteins encode fused structural and enzymatic viral proteins. Translation is regulated by two ribosomal frameshift events, leading to the production of Gag (87%), Gag-Pro (12%), and Gag-Pro-Pol (1%) (26). This two-frameshift strategy ensures that the Gag-Pro and Gag-Pro-Pol polyproteins encode G-patch motifs (Fig. 1 *B*, *Top* panel). In Gag-Pro, the G-patch (GP) is fused to protease (PR), while in Gag-Pro-Pol, the G-patch forms a connecting region between protease and RT. Following the packaging of gRNA into immature particles in the pericentriolar region, these retroviral polyproteins are transported to the plasma membrane and exit the host cell. During maturation, the viral protease cleaves the polyprotein precursors. Specifically, Gag is cleaved into major structural proteins (matrix MA, phosphoprotein pp, p12, capsid CA, nucleocapsid NC, and p4), Pro yields dUTPase (dUTP) and protease, and Pol maturation produces RT and IN (Fig. 1*B*) (27–29). The fate of the G-patch following virus maturation remains unclear; likely, the G-patch from Gag-Pro partially remains a C-terminal part of PR and partially cleaves out. The role of the G-patch in Gag-Pro-Pol is not understood. Although the significance of the G-patch motif for M-PMV infectivity has been previously reported (30, 31), its specific functions in immature or mature virions have yet to be elucidated.

In this study, we uncover a key interaction between the cellular helicase DHX15 and the retroviral G-patch, revealing a different mechanism by which M-PMV exploits host cellular mechanisms to facilitate its replication. Using a variety of advanced techniques, including interactome analysis, mutagenesis, RNA interference, CRISPR-Cas editing, and structural analysis, we show that the retroviral G-patch functions as a cofactor that specifically recruits DHX15 to viral particles. This binding mirrors the interaction patterns observed for cellular G-patch proteins and DHX15. Our results further demonstrate that retroviral G-patch with bound DHX15 is required for efficient gRNA packaging into the virion. Once inside the virions, the DHX15–G-patch complex is then essential for reverse transcription and influences the progression of reverse transcription as well as viral RT activity. We provide detailed experimental evidence that DHX15 recruitment and relocalization from the nucleus to the viral assembly site in the host cell cytosol is critical for the viral life cycle. In conclusion, our work significantly advances the understanding of the complex interplay between viral components and host cell machinery and opens different ways for exploring mechanisms of viral replication and potential therapeutic targets.

## Results

### Retroviral G-Patch Recruits Cellular DEAH/RHA-Box Helicase Into M-PMV Particles.

Similar to other retroviruses, M-PMV does not encode any intrinsic helicase and this activity relies entirely on host-cell enzymes. Because reverse transcription occurs within the protected environment of the viral core, it is likely that M-PMV must recruit and package cellular helicase into the viral particle, similar to HIV-1 (32). Given the anticipated importance of helicase activity for completing reverse transcription during the early stages of M-PMV infection, we analyzed the viral–host cell interactome. M-PMV, propagated in human embryonic kidney 293 (HEK 293) cells, was isolated and purified by ultracentrifugation using a combination of a sucrose cushion and an iodixanol gradient (Fig. 1 *C*, *Top* panel) as previously described (33). The presence of the virus was assessed by TEM (Fig. 1*D*). Mass spectrometry (MS) analysis of virus samples from three biological replicates showed the presence of 262 cellular proteins (Dataset S1); 60 of these were also identified in noninfected control samples. To exclude nonspecifically packaged proteins, we only considered those identified in all nine samples of purified M-PMV (three biological replicates, each measured in three technical replicates) and not identified in any of the three control samples. The resulting list of proteins stably packed into M-PMV particles is provided in *SI Appendix*, Table S1.

Interestingly, we identified DHX15, a member of the DEAH/RHA RNA helicase family, among these proteins. Since the G-patch motifs in several eukaryotic proteins function as cofactors of DHX15 (34–39), we hypothesized that the virus-encoded G-patch might function in a similar way. To explore whether DHX15 incorporation into M-PMV is mediated exclusively by the viral G-patch motif, we purified virus particles lacking G-patch (ΔGP M-PMV) (Fig. 1 *B* and *C*, *Bottom* panels), constructed as previously described (31). We analyzed the host-cell protein content in ΔGP M-PMV particles by MS and compared it with that of the wild-type (wt) M-PMV particles (Dataset S1). This comparison revealed that levels of some host-cell proteins were significantly reduced in ΔGP M-PMV compared to wt M-PMV. Notably, DHX15 level was most affected by the G-patch deletion (Fig. 1*E*). Immunoanalysis of purified wt and ΔGP M-PMV further confirmed that the G-patch deletion completely blocked incorporation of cellular DHX15 into viral particles (Fig. 1*F*). To rule out that this observation only applies to human cells, we also propagated M-PMV in African green monkey kidney cells (COS-1). Immunoanalysis of virus harvested from COS-1 cells confirmed the presence of DHX15 in wt M-PMV (Fig. 1*G*).

### Retroviral and Cellular G-Patch-Containing Proteins Have Similar Modes of Binding to DHX15.

**Fig. 2.**
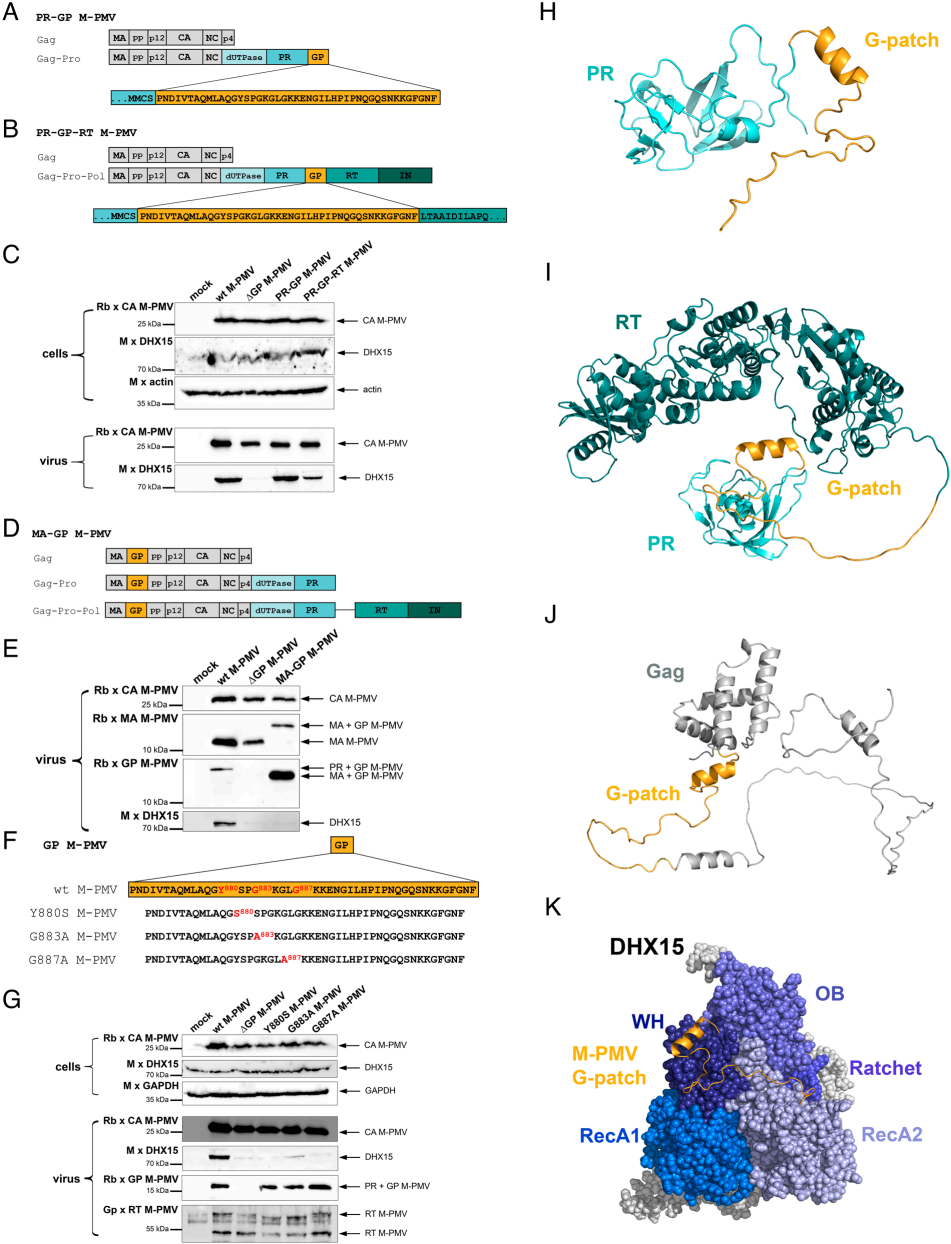
Mode of DHX15 binding to M-PMV G-patch. (*A* and *B*) Schematic representation of M-PMV frameshift variants: PR-GP M-PMV producing Gag and Gag-Pro (*A*) and PR-GP-RT M-PMV producing Gag and Gag-Pro-Pol (*B*), with amino acid sequences of G-patch regions. (*C*) Western blot analysis of wt M-PMV, ΔGP M-PMV, and M-PMV frameshift mutants, produced and released from HEK 293 cells. Viral particles were harvested from the culture medium and visualized using antibodies against M-PMV CA and DHX15. Actin was used as a sample loading control. (*D*) Schematic representation of M-PMV Gag-containing G-patch: MA-GP M-PMV. (*E*) Western blot analysis of wt M-PMV, ΔGP M-PMV, and MA-GP M-PMV produced in HEK 293 cells. The culture media were ultracentrifuged and visualized using antibodies against M-PMV CA, MA, and G-patch, and DHX15. (*F*) Amino acid sequence of M-PMV G-patch showing the mutations (red) used in this study. (*G*) Western blot analysis of cell- and virus-associated samples from the production of wt M-PMV, ΔGP M-PMV, and M-PMV G-patch mutants in HEK 293 cells. Samples were prepared as described above. Virus detection was performed using antibodies against M-PMV CA, G-patch, RT, and DHX15. GAPDH was used as a sample loading control. (*H*) AlphaFold model of the PR-GP portion of the M-PMV Pro polyprotein precursor, with PR in cyan and GP in yellow. (*I*) AlphaFold model of the PR-GP-RT part of the M-PMV Pol polyprotein precursor, with PR in cyan, GP in yellow, and RT in dark green. (*J*) Alphamodel of MA-GP-phosphoprotein of the M-PMV Gag polyprotein, with MA and pp domains of Gag in gray and GP in yellow. (*K*) Space-filling AlphaFold model of the complex of M-PMV G-patch (yellow) and DHX15 (blue); showing the individual DHX15 domains RecA1, RecA2, Ratchet, WH, and OB.

Cellular proteins containing a G-patch invariably possess a single copy of the motif positioned within their intrinsically disordered regions. In addition, these proteins often have one or more RNA-binding motifs (18). The two retroviral G-patch-containing polyprotein precursors, Gag-Pro and Gag-Pro-Pol, comprise several structural and enzymatic proteins, some of which possess RNA-binding motifs. These include two zinc-finger motifs in the NC domain of Gag and another RNA-binding motif in the RT domain of Pol. To determine whether DHX15 is bound preferentially by the G-patch of one of these polyprotein precursors, we used M-PMV frameshift mutants (26) producing either Gag and Gag-Pro polyproteins (PR-GP M-PMV, Fig. 2*A*) or Gag and Gag-Pro-Pol (PR-GP-RT M-PMV, Fig. 2*B*). These M-PMV mutant viruses, as well as the wt and ΔGP M-PMV vectors, were ultracentrifuged through a sucrose cushion and analyzed by immunoblot along with the producing cells (Fig. 2*C*). Virus-associated DHX15 protein was detected in all viral variants containing G-patch verifying that the M-PMV G-patch, whether from Gag-Pro or Gag-Pro-Pol polyproteins, can function as the DHX15 binding partner. To determine whether increasing the relative abundance of the G-patch sequence relative to the viral polyproteins would proportionally increase DHX15 incorporation, we transferred the G-patch sequence from its original position to a sequence between the MA and the phosphoprotein domain of the Gag polyprotein (MA-GP M-PMV, Fig. 2*D*). Surprisingly, no DHX15 was detected in released MA-GP viruses (Fig. 2*E*).

The crystal structure of the NKRF G-patch/DHX15 complex revealed that the G-patch binds through its N terminus to the WH (winged helix) domain of DHX15 helicase and via its C terminus to the RecA2 domain (20). Thus, the G-patch acts like a brace holding these two mobile helicase segments together. In this structure, the N terminus of the G-patch folds into a short amphipathic alpha-helix (termed the G-patch brace helix), which continues into an extended conformation allowing the C terminus (referred to as the G-patch brace loop) to bind to RecA2 domain. The conserved glycine residues of the G-patch do not directly bind to DHX15 but rather ensure the flexibility required for proper binding (20). The binding mode of Ssp2 G-patch to the human DHX16 ortholog Prp2 is almost identical (21). To investigate whether the betaretroviral G-patch/DHX15 binding mode corresponds to that observed between NKRF G-patch and DHX15, we analyzed virions with substitutions in key, highly conserved G-patch amino acids. Specifically, we analyzed the following substitutions downstream to the brace helix that align with crucial residues in NKRF G-patch: M-PMV Y880 (equivalent to W564 in NKRF), M-PMV G883 (NKRF G567), and M-PMV G887 (NKRF G571) (Fig. 2*F*) (20). Immunochemical analysis confirmed that DHX15 presence in purified G-patch mutant viruses is significantly reduced (Fig. 2*G*).

To test whether the M-PMV G-patch motif, like other cellular motifs, is unstructured and capable of forming an N-terminal brace helix, we used a combination of spectroscopic measurements and AlphaFold prediction. As the G-patch motif can act independently without requirements for any other protein domains (20), we analyzed purified M-PMV G-patch peptide, amino acids 867–911 (Fig. 1*A* and *SI Appendix*, Fig. S1*A*), by CD and NMR. According to the far-UV CD spectrum at room temperature, M-PMV G-patch peptide had a predominantly unordered structure together with a relatively small contribution of β-structure (*SI Appendix*, Fig. S1*C*, black line). However, temperature-induced changes in the CD spectra (*SI Appendix*, Fig. S1*A*) showed a “turn-out” response to heating, which is characteristic of several intrinsically disordered proteins (IDPs) (40). A similar result was observed in a Heteronuclear Single Quantum Coherence (HSQC) NMR experiment using uniformly ^15^N-labeled G-patch peptide. The spectrum showed a narrow distribution of cross-peaks around 8 ppm of the ^1^H frequency, which is typical for intrinsically disordered proteins (IDPs) (*SI Appendix*, Fig. S1*B*). On the other hand, in the presence of 2,2,2-trifluoroethanol (TFE), a compound known to promote peptide helicity, the G-patch exhibited a substantial portion of α-helical content, as evidenced by negative spectral bands at ~206 nm and ~222 nm (*SI Appendix*, Fig. S1*C*, red line). Next, we used AlphaFold prediction to build a 3D model of the structural arrangement of the M-PMV G-patch between PR and RT. Based on the NMR structure of M-PMV PR and structural analogy to other retroviral RTs, the AlphaFold-built models showed the G-patch positioned at the C terminus of the PR (Fig. 2*H*) and inside an unstructured part between the two well-structured PR and RT domains (Fig. 2*I*). In these models, the N terminus of G-patch forms a short alpha-helix in a position corresponding to the brace-helix observed in the NKRF G-patch/DHX15 and Ssp2 G-patch/Prp2 crystal structure (20, 21). The model with the G-patch inserted between the MA and PP domains of Gag showed no steric hindrance that could prevent DHX15 binding (Fig. 2*J*). However, the presence of C-terminal helix IV of the MA domain, which is only unfolded upon myristoyl switch (41), may reduce the flexibility of the N-terminal G-patch portion and compromise DHX15 binding. Nevertheless, AlphaFold analysis confirmed that the M-PMV G-patch peptide can adopt a conformation and binding mode with DHX15 similar to that of cellular NKRF G-patch (Fig. 2*K*), with a confidence more than 90% (*SI Appendix*, Fig. S1*D*). Taken together, these results suggest that the mode of interaction between the retroviral G-patch inside the Gag-Pro or Gag-Pro-Pol polyproteins and cellular DHX15 is similar to that described for cellular G-patch-containing proteins.

### DHX15 Is Essential for M-PMV Infectivity.

**Fig. 3.**
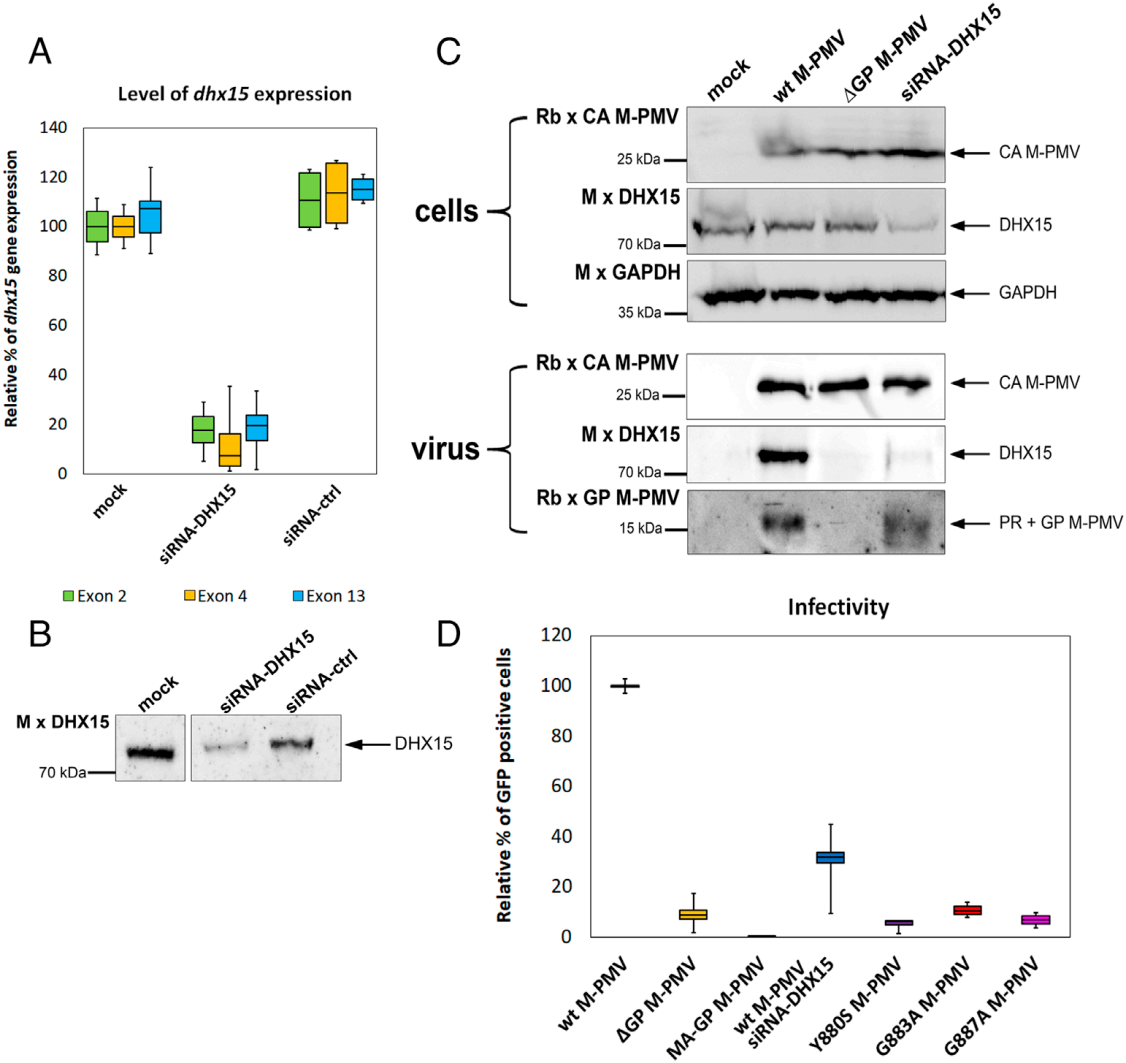
Downregulation of *dhx15* expression by siRNA. (*A* and *B*) HEK 293 cells were transfected with siRNA-DHX15 twice over 48 h. Samples were split for RNA isolation and qPCR quantification (*A*), and immunoanalysis with anti-DHX15 antibody (*B*). (*C*) HEK 293 cells, treated as in (*A* and *B*), were transfected with wt M-PMV. Virus production was compared to wt and ΔGP M-PMV. At 48 h posttransfection, viral particles were harvested and analyzed immunochemically using antibodies against M-PMV CA, GP, and DHX15. GAPDH was used as a loading control. (*D*) Relative infectivity of wt, ΔGP, and G-patch mutant M-PMV in HEK 293 cells and wt M-PMV in siRNA-DHX15-treated cells was assessed using a single-round infectivity assay. Virions harvested at 48 h posttransfection were used to infect HEK 293 cells. The amount of released virions was normalized to CA by the ELISA. After 48 h, infectivity was measured using a flow cytometer (BD FACS AriaIII).

To confirm the importance of DHX15 in the M-PMV replication cycle, we down-regulated DHX15 expression with specific siRNA. qPCR analysis revealed that DHX15 mRNA levels were reduced to 10 to 20% compared to untreated cells or those treated with control siRNA (Fig. 3*A*). The reduction of *dhx15* expression was further confirmed by western blot using monoclonal antibodies (Fig. 3*B*). Accordingly, immunoanalysis of M-PMV particles released from siRNA-treated cells showed a significant decrease in incorporated DHX15 compared to untreated cells (Fig. 3*C*). The infectivity of ELISA-normalized M-PMV released from siRNA-treated cells dropped significantly to roughly 30% of the infectivity of virus released from untreated cells (Fig. 3*D*). Similarly, the infectivity of M-PMV virions either bearing a G-patch deletion, Gag-insertion, or containing single-point substitutions of conserved G-patch residues were significantly impaired (Fig. 3*D*). These results correspond with previously published data (30, 31) reporting that G-patch deletion or substitutions of its conserved glycines severely diminish M-PMV infectivity. Since a similar decrease in infectivity was observed in DHX15 knockdown cells, these data further confirm the importance of the DHX15/G-patch interaction for M-PMV replication.

### DHX15 Colocalized with MPMV Near the Nucleus.

**Fig. 4.**
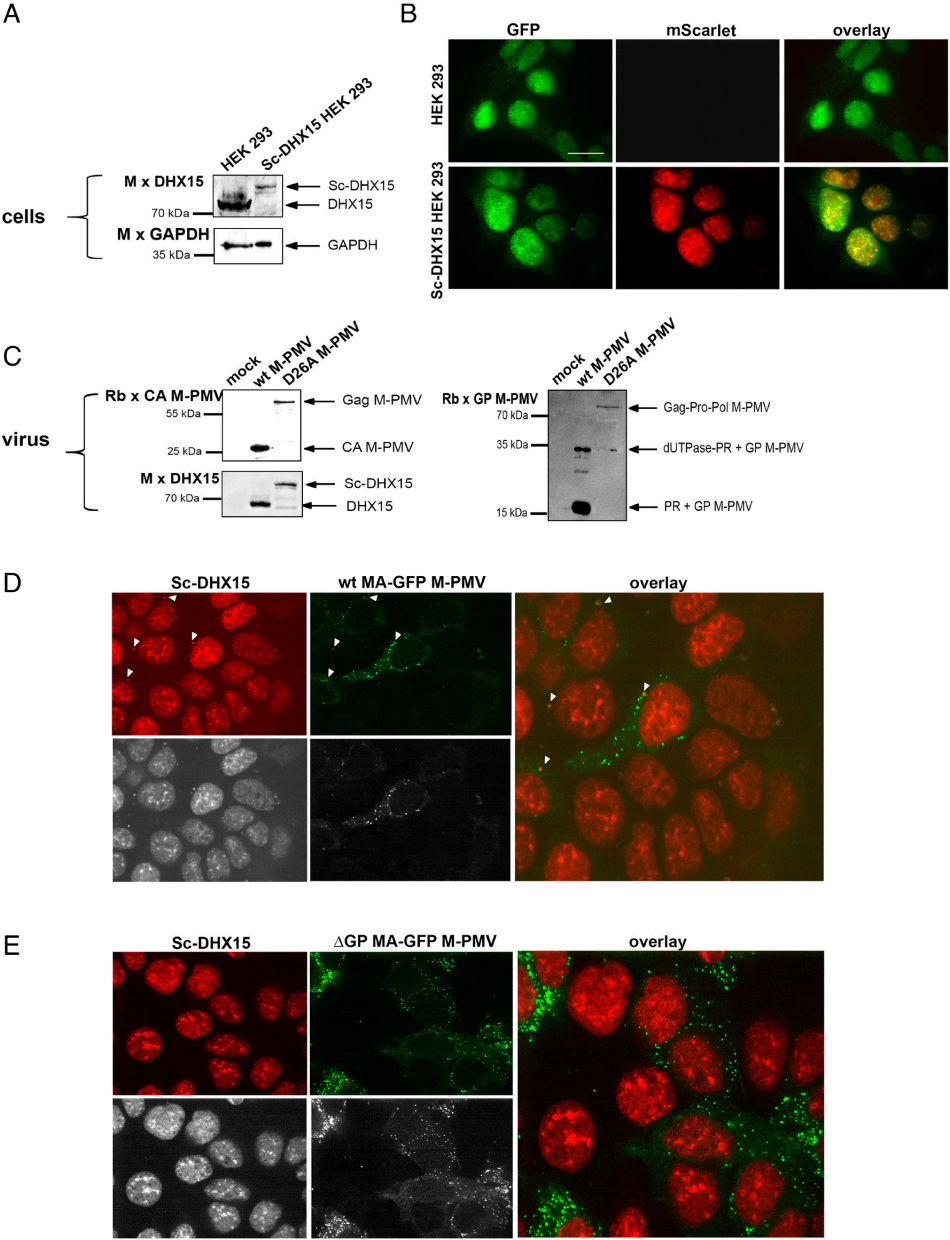
Colocalization of DHX15 with wt MPMV. (*A*) Immunochemical validation of Sc-DHX15 production in HEK 293 cells: HEK 293 cells were genetically engineered using CRISPR-Cas9 technology to express DHX15 with an N-terminal fusion to the mScarlet. Sc-DHX15 expression was confirmed by mouse antibody against DHX15, with GAPDH used as a sample loading control. (*B*) The subcellular localization of the DHX15 (*Top* panels) and Sc-DHX15 (*Bottom* panels) was analyzed by confocal fluorescent microscopy, using Rb × DHX15 and Alexa Fluor 488 goat anti-rabbit IgG. The bar represents 20 µm. (*C*) Western blot analysis of M-PMV particles produced from Sc-DHX15 HEK 293 cells: Particles were harvested 48 h posttransfection and identified using antibodies against the M-PMV CA, GP, and DHX15. The panel compares wt M-PMV with a protease-inactivated mutant (D26A), showing the cleavage of the GSGSGS linker by the viral protease. (*D* and *E*) Confocal microscopy analysis of Sc-DHX15 (red) and (*D*) wt MA-GFP M-PMV (green) and (*E*) ΔGP MA-GFP M-PMV (green) in Sc-DHX15 HEK 293 cells. Twenty-four hours posttransfection, the cells were fixed and imaged with a spinning disc confocal microscope. Spots of colocalization, observed only for wt M-PMV, are shown by white arrows in (*D*). In cells transfected with wt MA-GFP M-PMV, an average of 2.05 colocalization spots (SD 0.88) were counted. In comparison, cells transfected with ΔGP MA-GFP M-PMV showed an average of 0.13 spots (SD 0.18). The difference was statistically significant with a *P*-value < 0.00001 (Mann–Whitney *U* test).

DHX15 is primarily localized in the cell nucleus, nuclear speckles, and nucleolus (42, 43). Interestingly, Prp43, the yeast homolog of DHX15, can be sequestered from the nucleus to the cytosol by increasing the expression of its G-patch interaction partner (42). Based on these observations, we hypothesized that DHX15 is incorporated into M-PMV virions through direct cytosolic interaction with the G-patch of the M-PMV Gag-Pro and Gag-Pro-Pol polyproteins. To investigate the colocalization of DHX15 with M-PMV G-patch-containing polyproteins, we generated an mScarlet-DHX15 HEK-293 cell line by using Crisper-Cas technology. The production of DHX15 and DHX15 with N-terminal fusion to fluorescent mScarlet protein (Sc-DHX15) was verified through immunochemical analysis (Fig. 4*A*). Potential effect of N-terminal mScarlet addition to DHX15 on its subcellular localization was analyzed by confocal fluorescence microscopy (Fig. 4*B*). Both DHX15 (Fig. 4 *B*, *Top* panels) and Sc-DHX15 (Fig. 4 *B*, *Bottom* panels) were predominantly localized in similar speckles-like structures, suggesting that the mScarlet tag does not significantly alter nuclear localization of DHX15. We observed that M-PMV protease cleaves the flexible GSGSGS linker between mScarlet and DHX15 during virion maturation. Utilizing a protease-inactivation mutant (D26A), we however verified the incorporation of Sc-DHX15 into M-PMV virions (Fig. 4*C*). Colocalization experiments were performed in the Sc-DHX15 HEK-293 cell line transfected with either wt or ΔGP M-PMV proviral vectors, in which GFP was inserted between MA and phosphoprotein, MA-GFP-M-PMV (Fig. 4*D*) and MA-GFP-ΔGP-M-PMV (Fig. 4*E*), respectively. For wt M-PMV, colocalization was indicated by the spectral overlap of red and green fluorescence, which was particularly evident in the gray-scale images (Fig. 4*D*). In wt M-PMV producing Sc-DHX15 cells, a single prominent colocalization spot per cell was usually observed (highlighted by white arrowheads Fig. 4*D*). The intracellular localization of these individual spots within the cells—close to the nucleus—may indicate potential viral assembly sites, which for M-PMV is represented by the pericentriolar site. No such cytosolic localization of Sc-DHX15 was observed either in noninfected cells (Fig. 4*B*) or in the cells producing ΔGP- M-PMV (Fig. 4*E*). The clear separation of the red and green signals with no apparent overlay in the merged image suggests a significant decrease in the colocalization between Sc-DHX15 and ΔGP- M-PMV. These results provide strong evidence for our hypothesis that the G-patch domain of the M-PMV Gag-Pro and Gag-Pro-Pol polyproteins is a necessary component for the interaction with DHX15, facilitating its incorporation into the assembling virion.

### DHX15 Is Required for Proper gRNA Packaging in a G-Patch-Dependent Manner.

**Fig. 5.**
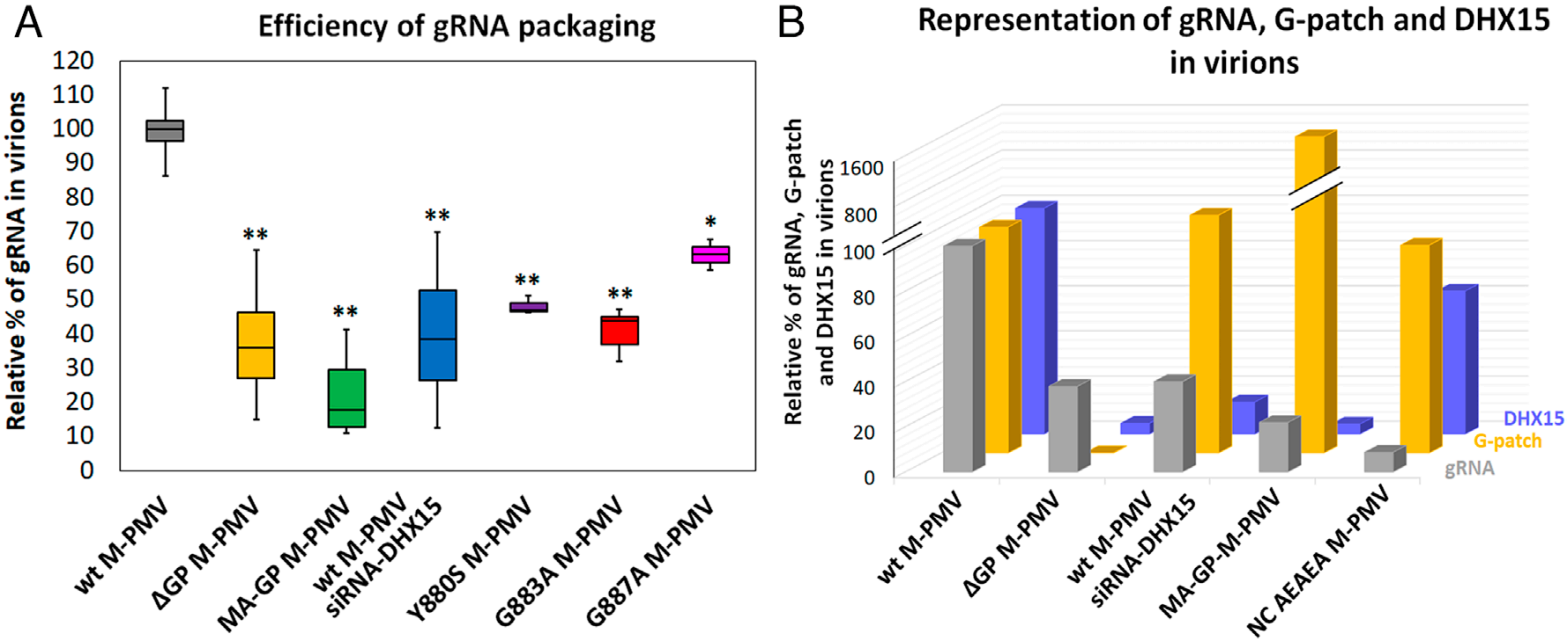
DHX15 affects M-PMV genomic RNA packaging. (*A*) The gRNA content in G-patch mutant viruses and wt M-PMV released from HEK 293 cells treated with siRNA-DHX15 was determined and calculated relative to wt M-PMV. Virions were isolated from culture media 48 h posttransfection with proviral DNA. Viral RNA was isolated from viral particles following normalization by the ELISA and purification by ultracentrifugation through a 20% sucrose gradient, and M-PMV gRNA was quantified by RT-qPCR. (*B*) Quantification of virus-associated G-patch and DHX15 based on Image LabSoftware western blot analysis of M-PMV particles released from HEK 293 cells, and gRNA based on quantified by RT-qPCR.

Unexpectedly, analysis of the released M-PMV particles revealed a significantly reduced content of viral gRNA incorporated into ΔGP M-PMV compared to wt virions (Fig. 5*A*). To validate this finding, we also quantified the gRNA content in other mutant M-PMV virions and in virions released from siRNA-DHX15-treated cells. A significant reduction in gRNA content was observed in all particles in which either the presence or functionality of the G-patch or DHX15 was compromised (Fig. 5*A*). Since the mutations in the G-patch or reduced expression of DHX15 cannot affect the function of nucleocapsid (NC) in the Gag polyprotein, which is the primary domain responsible for gRNA recognition and binding, our results suggest that successful gRNA packaging into viral particles also depends on both the G-patch and DHX15.

To better understand the interplay between M-PMV G-patch, DHX15, and gRNA, we quantified the amount of virus-associated G-patch, DHX15, and gRNA (Fig. 5*B*). The absence of the G-patch resulted in undetectable DHX15 and a reduction of gRNA to 40% of the wt level. A similar decrease in gRNA was detected when DHX15 was down-regulated by si-RNA knockdown, despite the wt-level of G-patch. Surprisingly, gRNA levels were significantly reduced to 20% of wt in the MA-GP mutant, which produces ten times more G-patch than wt, but is unable to bind DHX15 (Fig. 5*B*). Finally, using an NC mutant AEAEA-M-PMV (44), which has an impaired ability to bind gRNA, we found that despite containing wt levels of both G-patch and DHX15, gRNA incorporation was not rescued. This result clearly demonstrates that although the M-PMV G-patch/DHX15 complex is important for gRNA packaging, without the primary gRNA recognition and binding signal provided by NC, the potential secondary gRNA packaging signal mediated by G-patch and DHX15 is ineffective (Fig. 5*B*). These results demonstrate that the DHX15–G-patch interaction function as an additional, however essential signal for efficient gRNA packaging.

To confirm the potential interaction of DXH15 with M-PMV gRNA, we employed photoactivatable-ribonucleoside-enhanced cross-linking and immunoprecipitation (PAR-CLIP). M-PMV-transfected cells grown in the presence of 4-thiouridine were exposed to UV light. The RNP complexes from the cytosolic fraction were immunoprecipitated using an anti-DHX15 monoclonal antibody, and isolated RNA was reverse transcribed and sequenced. The results showed that DHX15 interacts specifically with the 7,328 to 7,393 bp of M-PMV gRNA, which includes the CTE and its upstream region (*SI Appendix*, Fig. S2).

### DHX15 Participates in Reverse Transcription.

**Fig. 6.**
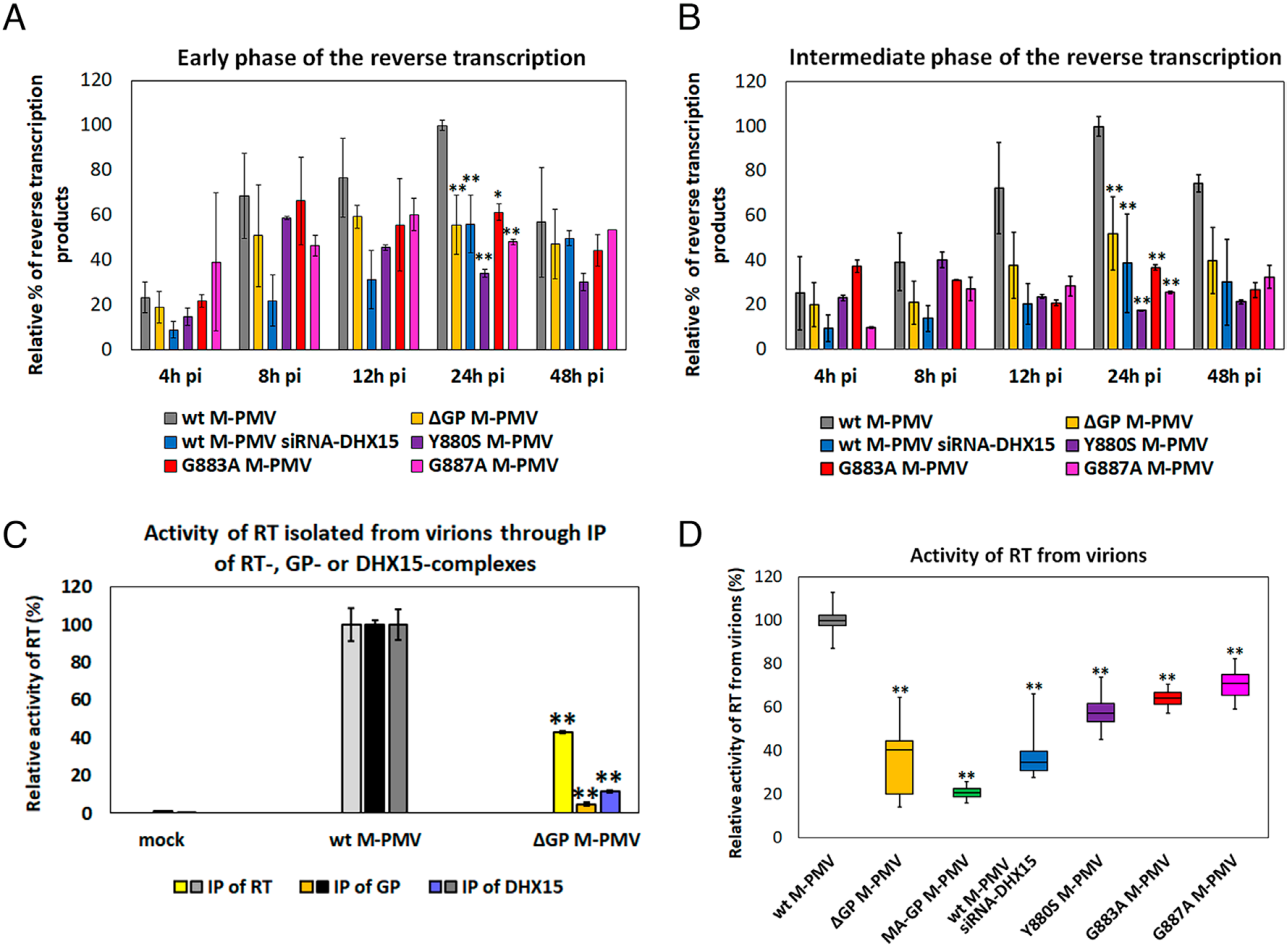
DHX15 is part of the reverse transcription complex in mature M-PMV particles. (*A* and *B*) Reverse transcription of wt and GP mutant M-PMV produced in HEK 293 cells and wt M-PMV produced in siRNA-DHX15-treated HEK 293 cells. To monitor reverse transcription, HEK 293 cells were infected with wt and mutant M-PMV and harvested at 4, 8, 12, 24, and 48 h postinfection. Real-time PCR analysis of isolated DNA was used to detect reverse transcription products of the early (*A*) and intermediate (*B*) phases. The results were normalized using the ELISA and housekeeping genes. To compare all biological replicates, the normalized amount of DNA was calculated as the % of products relative to the maximum of wt M-PMV at 24 h postinfection. (*C*) Determination of RT activity in immunoprecipitated complexes. Wt and ΔGP M-PMV were concentrated by ultracentrifugation, and GP and DHX15 complexes were immunoprecipitated. RT activity of the immunoprecipitated complexes was assessed by RT-qPCR and calculated as relative % directly proportional to the RT-qPCR products. (*D*) Activity of M-PMV RT. Wt, ΔGP, and GP mutant M-PMV were produced in HEK 293 cells. Wt M-PMV was also produced in siRNA-DHX15-treated HEK 293 cells. Virions were normalized by the ELISA, and RT activity was determined by the colorimetric assay. All *P* values were assessed by ANOVA with a combination of the Tukey–Kramer HSD test and the Scheffé, Bonferroni, and Holm multiple comparison tests. *P* values (**P < 0.01 and *P < 0.05) were calculated by comparing samples to wt M-PMV, unless otherwise stated.

Apart from the unexpected role of DHX15 in gRNA packaging, we tested its more anticipated role in RNA remodeling during reverse transcription in M-PMV virions. We compared the reverse transcription kinetics of virions released from siRNA-DHX15-treated HEK 293 cells; ΔGP M-PMV; Y880S, G883A, and G887A mutant virions released from siRNA-untreated HEK 293 cells; and wt M-PMV. At intervals of 4, 8, 12, 24, and 48 h postinfection, total DNA was isolated from cells infected with ELISA-normalized amounts of M-PMV virions and used for qPCR quantification of reverse transcription products of the early (Fig. 6*A*) and intermediate (Fig. 6*B*) phases. While the early phase is characterized by synthesizing strong-stop DNA, complementary minus-strand DNA is produced during the intermediate phase. Although the intermediate phase was more significantly impacted, there were substantial reductions in production of reverse transcription products during both phases in cells infected with ΔGP M-PMV, G-patch mutant virions, and virions produced in siRNA-treated cells. This decline was especially pronounced 24 h postinfection, coinciding with peak levels of reverse transcription products in cells infected with wt M-PMV.

To elucidate the effect of G-patch/DHX15 on reverse transcription, we investigated whether DHX15 is associated with the reverse-transcribing complex in mature virions. We isolated wt and ΔGP M-PMV virions and performed immunoprecipitation (IP) using antibodies against RT, G-patch, and DHX15, followed by an assessment of RT activity by RT-PCR assay (Fig. 6*C*). For wt M-PMV, RT activity was detected in samples immunoprecipitated with all antibodies, whereas significantly reduced RT activity was observed in the ΔGP M-PMV IP samples. These results strongly suggest that formation of the M-PMV G-patch/DHX15 complex is essential for RT activity and that RT, G-patch, and DHX15 are associated within mature virions. Finally, we investigated whether the interaction between DHX15 and G-patch plays a role in modulating M-PMV RT activity. Based on our previous findings indicating that G-patch affects the activity of M-PMV RT (31), we investigated how the G-patch mutations connected with lack of DHX15 incorporation into the virions affect RT activity. We used a colorimetric assay to measure the RT activity of wt, ΔGP, Y880S, G883A, and G887A M-PMV virions (Fig. 6*D*). Our results show that all mutants and virions produced in siRNA-DHX15-treated cells displayed reduced RT activity compared to wt M-PMV. It is important to note that the assays used for RT activity (colorimetric and RT-qPCR) utilized artificial RNAs and complementary primers, which do not recognize M-PMV gRNA. This ensures that the results of the measurements depicted in Fig. 6 *C* and *D* are not affected by the gRNA content. This implies that a complex of G-patch bound to DHX15 is essential for M-PMV RT activity. Furthermore, the DHX15 binding to G-patch-RT appears to be essential for optimal functioning of the RT complex, particularly during the early and intermediate phases.

## Discussion

This study reveals a broad aspect of retroviral replication and highlights the critical role of the cellular DEAH box RNA helicase DHX15 in the life cycle of Mason-Pfizer monkey virus. Our results show that the retroviral G-patch acts as a molecular beacon that specifically recruits DHX15 into the virion, by a mechanism that mirrors the interaction observed between cellular G-patch proteins and DHX15. Through a comprehensive approach, including mutagenesis, RNA silencing, CRISPR-Cas engineering, PAR-CLIP analysis, and structural analysis, we confirm that M-PMV G-patch-driven recruitment and relocalization of DHX15 from the nucleus to the cytosol is essential for efficient viral gRNA packaging. Significantly, we also show that the DHX15–G-patch complex in the virions plays a critical role in the progression of reverse transcription and the activity of viral RT.

Retroviruses lack intrinsic helicase activity and rely entirely on cellular helicases. Our study highlights a distinct strategy employed by M-PMV that distinguishes it from other retroviruses, such as HIV-1, which utilize a variety of cellular helicases. More than 20 DExD/H box RNA helicases, including DHX9, (also known as DDX9 or RNA helicase A) are critical for replication of HIV-1. These helicases mediate several processes, including viral RNP nuclear export, viral RNA splicing, viral transcription, and translation (for review, see refs. 24, 25, and 45). DHX9, can bind the NC domain of Gag and the 5’UTR region of HIV-1 genomic RNA and become incorporated into HIV-1 particles (19, 46) in a viral gRNA-dependent manner (47). Subsequently, DHX9 enhances HIV-1 infectivity by accelerating reverse transcription (20, 21). M-PMV, a simple retrovirus, adopts a strategy distinct from that of HIV-1. The selective recruitment of DHX15 through the unique G-patch motif highlights a sophisticated mechanism of host–pathogen interaction and reveals the evolutionary complexity of viral replication strategies. The absence of DHX15 in M-PMV virions, caused either by mutation(s) or deletion of the M-PMV G-patch motif or by downregulation of host cell DHX15 by siRNA, negatively affects packaging of gRNA into the viral particles, the progression of reverse transcription and it blocks virus infectivity. The impact of missing helicase activities, either DHX9 for HIV-1 (46) or DHX15 for M-PMV, in mature virions confirms that helicase activity associated with the mature retroviral core is essential for the proper function/activity of the reverse-transcribing complex in subsequently infected cells. The need for helicase incorporation into HIV-1 is strongly supported by the observation that reverse transcription of viral gRNA in newly infected cells occurs within the enclosed environment of the viral capsid core, which dissociates when reverse transcription is completed in the nucleus (for review, see ref. 48). Hence, to accomplish reverse transcription, both HIV-1 and M-PMV need to package helicase into newly formed virus particles.

Cellular DHX15 is incorporated into M-PMV particles by the retroviral G-patch, which resides within the two large polyprotein precursors Gag-Pro and Gag-Pro-Pol, with respective molecular weights of 95 and 180 kDa. In Gag-Pro, the G-patch motif is positioned at the very end of the polyprotein, downstream of the aspartic protease. Following a frameshift event that overcomes the stop codon at the 3′ end of the *pro* region, the G-patch becomes situated between PR and RT. Our AlphaFold model shows that the G-patch comprises an intrinsically disordered region that flanks the C terminus of the well-structured PR region in Gag-Pro (49), while in Gag-Pro-Pol, it connects PR with another well-structured RT domain. This configuration is reminiscent of eukaryotic G-patch proteins, enabling the G-patch to effectively “fish” for the helicase. Our CD, NMR, and AlphaFold data indicate that while the retroviral G-patch remains largely unstructured, it can fold into an N-terminal alpha helix known as the brace helix, which is crucial for DHX15 binding. Mutations targeted downstream of the G-patch brace helix, specifically those substituting conserved amino acid residues demonstrated to be critical for cellular NKRF G-patch binding to DHX15, play an important role in M-PMV. Specifically, W564 is essential for preserving the overall structure of the NKFR G-patch motif (20). Our experimental data revealed that the analogously positioned Y880 residue in M-PMV G-patch is indispensable for DHX15 binding. Our AlphaFold model indicates that M-PMV Y880 is a key residue for maintaining the G-patch structure due to the potential formation of a hydrogen bond between Y880 and the backbone amide group of G883 (*SI Appendix*, Fig. S1*E*). This bond is particularly important as it induces a bend in the downstream peptide, composed of conserved glycine residues, toward the DHX15 hydrophobic cavity. The AlphaFold model agrees with our experimental findings that mutations of Y880 and G883 completely abolish incorporation of DHX15. The model also shows the positioning of a conserved glycine (G887) toward a DHX15 cavity consisting of V522, H523, F524, F526, and P529 (*SI Appendix*, Fig. S1*E*), which is similar to the position of G570 in the experimentally determined structure of NKFR G-patch. Substitution of this residue with alanine strongly affected DHX15 incorporation. Taken together, the results of our mutational analysis of M-PMV G-patch indicate that the DHX15 binding mechanisms of retroviral G-patch-containing polyproteins and cellular G-patch-containing proteins are mediated by the same residues.

An intriguing aspect of our study is the finding that the formation of M-PMV G-patch and DHX15 complex is critical for efficient incorporation of gRNA into new viral particles. Retroviral gRNA is synthesized in the nucleus, and retroviruses encode cis-acting nuclear export RNA elements that allow the transport of unspliced, intron-containing gRNA that would otherwise remain sequestered in the nucleus. HIV-1 uses a Rev response element (RRE) in combination with the cellular nuclear export receptor CRM1, while simple retroviruses such as M-PMV encode a constitutive transport element (CTE) that recruits components of the nuclear export machinery mRNA NXF1(Tap)/NXT1(p15), reviewed in ref. 50. Direct visualization of nucleocytoplasmic transport of retroviral gRNAs suggested that RNA export elements play a role in distributing gRNAs in the cytoplasm. The HIV-1 RRE regulates the translocation of mRNAs from the nucleus to the cytoplasm in a “burst-like” nonlocalized manner, whereas the M-PMV CTE associates gRNAs with microtubules and controls their distinct clustering close to a microtubule-organizing center (MTOC) (51). Although the packaging of gRNA by retroviruses is essential for infectivity, the subcellular site of the initial interaction between the viral polyprotein and gRNA remains poorly defined. In the case of C-type retroviruses (HIV, Rous sarcoma virus, RSV) that assemble at the plasma membrane, it was thought that viral polyproteins initially bind to gRNA in the cytoplasm or at the plasma membrane. However, RSV Gag protein has been shown to be actively transported to the nucleus where it binds to and exports unspliced viral RNA, possibly for packaging into virions as viral genome (52–54). M-PMV as a D-type retrovirus assembles intracytoplasmic particles deep in the cytosol, close to the nucleus (55).

The very interesting point is the specific intracellular place where DHX15 interacts with M-PMV G-patch. Since the synthesis of MPMV polyproteins and the subsequent assembly of viral particles take place in the cytosol, this interaction was expected to occur in the cytoplasm. However, our data showing that Sc-DHX15 is primarily localized to the nucleus and revealing an interaction between DHX15 and M-PMV gRNA region upstream of CTE, a critical RNA element for nuclear export of unspliced viral RNA (56), makes the situation far more intriguing. Since the G-patch functions as a DHX15 cofactor, activating and regulating helicase function, the G-patch-containing M-PMV Gag-Pro or Gag-Pro-Pol probably has to be transported to the nucleus for binding and activation of DHX15. This complex must then be exported from the nucleus to the site of viral particle assembly. Since we did not observe a decrease in gRNA-driven viral polyprotein expression, it is likely that DHX15 does not directly affect nuclear export of gRNA to the cytosol. The need of M-PMV G-patch-activated DHX15 helicase to bind gRNA CTE may therefore be in the remodeling of gRNA either required for gRNA dimerization or for efficient packaging. The ability to remodel RNP complexes has been reported for several RNA helicases, including DHX15 (for review, see ref. 14).

The interplay between G-patch proteins and DEAH-box helicases is key to numerous cellular processes, including RNA remodeling, RNA splicing, cell differentiation and innate immunity (7, 10, 15). Our study adds to the understanding of the versatility of DHX15. It also raises the intriguing possibility that DHX15’s modulation of host mRNAs containing constitutive transport elements (CTEs) is mediated by G-patch motif-containing cellular proteins. A particularly compelling example is the identification of a functional CTE within the alternatively spliced intron 10 of the NXF1/TAP gene, a critical component of the NXF1(Tap)/NXT1(p15) complex that facilitates general mRNA nuclear export (57). The translation of this CTE-NXF1 transcript into a truncated form of NXF1 implies a self-regulatory mechanism affecting its own expression. This finding positions CTEs as important not only for retroviral gRNA export but also in the broader context of mammalian mRNA export regulation, particularly of intron-retaining mRNAs (IR-mRNAs). As IR-mRNAs have been implicated in several diseases, including neurodegenerative disorders and cancer (58, 59), the potential regulation of these mRNAs by DHX15, possibly through interactions with cellular G-patch proteins, opens different ways of research that could lead to innovative therapeutic interventions.

In conclusion, these findings provide a deeper understanding of the molecular intricacies of retroviral replication and host–pathogen interactions and lay the base for future research into viral exploitation of host RNA processing pathways.

## Materials and Methods

### Expression Vectors.

All DNA manipulations were carried out using standard subcloning techniques, and plasmids were propagated in *Escherichia coli* DH5α. All newly created constructs were verified by DNA sequencing. The proviral M-PMV pSARM4 vector as well as the pSARM-EGFP and pTMO vectors were kindly provided by Eric Hunter (Emory University). As previously described for HIV MA-GFP (60), the GFP sequence was inserted between MA and phosphoprotein sequences to create two vectors wt MA-GFP MPMV and ΔGP MA-GFP M-PMV. All mutations in the M-PMV G-patch were introduced as previously described (31). To produce the M-PMV G-patch in bacterial cells, a vector GST-TEV-GP/pET41a was constructed by cloning a PCR fragment encoding the M-PMV G-patch into pET41a using *Nhe*I and *Xho*I, in frame with the upstream GST-TEV region. The His-3C-DHX15 pFAST Bac vector, encoding His-DHX15, was kindly provided by Umeharu Ohto (University of Tokyo, Japan).

### Cell Cultures.

HEK 293 and COS-1 cells were grown in Dulbecco’s modified Eagle medium (DMEM, Sigma) supplemented with 10% fetal bovine serum (Sigma) and 1% L-glutamine (Sigma) at 37 °C under 5% CO_2_. Typically, 1 d before transfection, cells were plated at a density of 2 to 5 × 10^5^ cells/mL. The following day, cells were transfected with an appropriate plasmid using either polyethylenimine (PEI) or X-tremeGENE HP (Roche) with a 1:2 or 5:1 ratio of DNA:transfection agent, respectively. The cells were grown for an additional 6 to 48 h and then used in subsequent experiments.

### Single-Round Infectivity Assay.

The infectivity assay was performed as previously described (61, 62). Briefly, at 48 h posttransfection, medium containing M-PMV virions was collected and filtered through a 0.45 µm filter. Freshly seeded HEK 293 cells were infected with ELISA-normalized amounts of M-PMV and incubated. After 48 h, the cells were harvested, fixed with 4% paraformaldehyde, and transferred to a FACS tube. Quantification of GFP-positive cells was performed using a BD FACS AriaIII flow cytometer.

### M-PMV Virion Production.

HEK 293 cells were transfected with the proviral M-PMV vector pSARM4. At 48 h posttransfection, the culture medium containing released virions was filtered through a 0.45 µm filter and ultracentrifuged through a 20% sucrose cushion at 210,000×*g* for 1 h in a Beckman SW41Ti rotor. The viral pellets were then resuspended in a buffer suitable for subsequent experiments.

### Isolation and Purification of M-PMV Virions, MS Analysis, and Label-Free Protein Quantification.

Virions were produced and isolated as described previously (33), for details, see *SI Appendix*. MS analysis was performed on samples of wt and ΔGP M-PMV virions, each in three biological replicates (obtained from three independent isolations of virions) and three technical replicates. Noninfected cells served as control samples and were also analyzed in three biological replicates. Refer to *SI Appendix* for a detailed description of sample preparation, MS analysis, and the label-free quantification procedure.

### Immunoblotting.

Proteins were resolved by reducing SDS-PAGE and blotted onto a nitrocellulose membrane. Nonspecific interactions were blocked with Blocking Solution (Thermo Scientific). The antigen–antibody complexes were detected by Clarity™ Western ECL (Bio-Rad) or West Atto Chemiluminescent Substrate (Thermo Scientific) using a Quantum Vilber Lourmat imager (Schoeller). The antibodies used in the study were as follows: Rbx M-PMV CA, Rbx M-PMV PR, Rbx M-PMV G-patch (prepared in house), MxDHX15, Mx actin, Mx GAPDH (obtained from SCBT, TX), GxRb HRP, Rbx M HRP (obtained from Sigma, MO).

### TEM.

Purified M-PMV particles were negatively stained using 2% sodium phosphotungstate (pH 7.4) on carbon-coated grids, as previously described (63, 64). The samples were visualized by TEM using a JEOL transmission electron microscope JEM-1010 at 80 kV. Images were captured using an AnalySIS MegaView III digital camera.

### Protein Expression and Purification.

GST-TEV-GP and His-3C-DHX15 were produced in bacterial cell *E. coli* BL21(DE3) and insect cells Sf9, respectively. Following production, the cells were lysed and the proteins were purified using the AKTA protein purification chromatography system, by a combination of affinity and size-exclusion chromatography. Further details on protein purification can be found in *SI Appendix*.

### Circular Dichroism (CD).

CD spectra were measured on a Jasco 1500 spectropolarimeter equipped with a Peltier thermostated holder PTC-517. ECD spectra in the far-UV spectral region (195 to 280 nm) were obtained over a temperature range from 10 °C to 90 °C with 10 °C steps. The sample concentration was 0.11 mg/mL in a quartz cell with 0.5 mm path length. Details for the experimental setup can be found in *SI Appendix*.

### NMR Spectroscopy.

2D ^1^H-^15^N HSQC experiments were measured at 298 K on a Bruker Avance III 600 MHz NMR spectrometer equipped with a cryoprobe. NMR data were processed with TopSpin 3.5 (Bruker BioSpin, GmbH) and further analyzed with the CcpNmr Analysis 2.5.2 program (65).

### siRNA Knock-Down.

The experiments were performed as previously described (66). Briefly, siRNAs targeting DHX15 were purchased from ORIGENE, and their impact on DHX15 expression was evaluated by RT-qPCR and immunoanalysis. For further details, refer to *SI Appendix*.

### Analysis of *dhx15* Gene Expression Levels Using RT-qPCR.

Expression of *dhx15* was determined by detecting exons 2, 4, and 13 by qPCR in isolated and reversely transcribed cellular mRNA. Phospholipase A (PLA) and glyceraldehyde-3-phosphate (GAPDH) were used as reference genes. qPCRs were performed using a QuantStudio™ 5 Real-Time PCR System. Additional details are provided in *SI Appendix*.

### qPCR Analysis of Reverse Transcription Products.

The products of the early and intermediate phases of reverse transcription were quantified by targeting the strong-stop DNA and *gag*-derived gene encoding M-PMV phosphoprotein, respectively, as detailed previously (44, 67, 68). Total DNA from HEK 293 cells infected with ELISA-normalized M-PMV was isolated at different time points postinfection using a DNeasy Blood & Tissue Kit (Qiagen). The target gene was then detected by qPCR using 2× SYTO-9 Master Mix (Top-Bio) supplemented with 1 µM M-PMV strong-stop DNA. *PLA* and *GAPDH* were used as reference genes. The pSARM-EGFP vector carrying M-PMV genome was used to establish a calibration curve in the 1.18 to 4,840 pg/µL concentration range. RT products were quantified by a standard curve-based method and normalized to reference gene expression.

### Analysis of gRNA Packaging Efficiency.

M-PMV gRNA packaging into virions was determined by RT-qPCR as previously described (44). Briefly, 48 h after transfection, a cultivation medium containing M-PMV was harvested, filtered, quantified by the ELISA, and ultracentrifuged through a 20% sucrose cushion. Viral gRNA was isolated using the QIAamp Viral RNA Mini kit (Qiagen) followed by TURBO DNase (Invitrogen™) treatment. M-PMV gRNA was then detected by RT-qPCR using *gag*-specific primers. The relative amount of packaged gRNA was calculated using the ΔΔCq method.

### Activity of RT.

This assay was conducted as previously described (44). Briefly, viruses from the culture media were collected 48 h posttransfection, filtered through 0.45 µm filters, and concentrated by ultracentrifugation through a 20% sucrose cushion as described above. The M-PMV RT activity in individual samples was then determined by the RT Colorimetric Assay (Sigma-Aldrich), including synthetic RNA template and corresponding primers, using an Infinite® 200 PRO series spectrophotometer (Tecan M200) at 490 nm.

### Determination of RT Activity After IP through GP and DHX15 Antibodies.

Virions were pelleted through a 20% sucrose cushion, and the resulting pellet was resuspended in RT lysis buffer and incubated with antibody against DHX15 or M-PMV G-patch, and then with magnetic beads conjugated with Protein A. RT activity was determined by RT-qPCR using artificial target SLA RNA with appropriate primers. The full experimental details can be found in *SI Appendix*.

### Generation of mScarlet-DHX15 Producing HEK 293 Cell Line.

The mScarlet-DHX15 HEK-293 cell lines were prepared using CRISPR-Cas9 technology. The gRNA was then isolated and sequenced from the selected clones to confirm the specific genome editing. The expression of the Sc-DHX15 protein was detected immunochemically using the MxDHX15 antibody (SCBT) and by fluorescence microscopy. Further details are provided in *SI Appendix*.

### Fluorescence Microscopy.

mScarlet-DHX15 HEK 293 cells were grown on 35 mm dishes with glass bottom (MatTek) and transfected with wt MA-GFP M-PMV and ΔGP MA-GFP M-PMV proviral vectors. Twenty-four hours posttransfection, the cells were fixed in 4% paraformaldehyde in PBS at room temperature and imaged with a spinning disc confocal microscope (Andor, Belfast, UK). For further details regarding the experimental and statistical analysis, refer to *SI Appendix*.

### PAR-CLIP-seq and Bioinformatic Analyses.

The PAR-CLIP-seq experiments were based on a previously published protocol (69), with some modifications. Protein binding sites were identified using the nf-core/clipseq pipeline. The genome coverage was calculated using the SAMtools (70). For details regarding PAR-CLIP and the associated bioinformatic analysis, see *SI Appendix*.

## Supplementary Material

Appendix 01 (PDF)

Dataset S01 (XLSX)

## Data Availability

All study data are included in the article and/or supporting information. The mass spectrometry proteomics data have been deposited to the ProteomeXchange Consortium via the PRIDE partner repository with the dataset identifier PXD046672 (71).

## References

[1] C. F. Bourgeois, F. Mortreux, D. Auboeuf, The multiple functions of RNA helicases as drivers and regulators of gene expression. Nat. Rev. Mol. Cell Biol. **17**, 426–438 (2016). 27251421 10.1038/nrm.2016.50

[2] J. C. Marecki, B. Belachew, J. Gao, K. D. Raney, RNA helicases required for viral propagation in humans. Enzymes **50**, 335–367 (2021). 34861942 10.1016/bs.enz.2021.09.005 PMC8562938

[3] M. E. Fairman-Williams, U.-P. Guenther, E. Jankowsky, SF1 and SF2 helicases: Family matters. Curr. Opin. Struct. Biol. **20**, 313–324 (2010). 20456941 10.1016/j.sbi.2010.03.011 PMC2916977

[4] M. R. Singleton, M. S. Dillingham, D. B. Wigley, Structure and mechanism of helicases and nucleic acid translocases. Annu. Rev. Biochem. **76**, 23–50 (2007). 17506634 10.1146/annurev.biochem.76.052305.115300

[5] A. M. Pyle, Translocation and unwinding mechanisms of RNA and DNA helicases. Ann. Rev. Biophys. **37**, 317–336 (2008). 18573084 10.1146/annurev.biophys.37.032807.125908

[6] J. Robert-Paganin , Functional link between DEAH/RHA helicase Prp43 activation and ATP base binding. Nucleic Acids Res. **45**, 1539–1552 (2017). 28180308 10.1093/nar/gkw1233 PMC5388414

[7] J. Robert-Paganin, S. Rety, N. Leulliot, Regulation of DEAH/RHA helicases by G-patch proteins. BioMed Res. Int. **2015**, 931857 (2015). 25692149 10.1155/2015/931857 PMC4322301

[8] M. J. Tauchert, J.-B. Fourmann, R. Lührmann, R. Ficner, Structural insights into the mechanism of the DEAH-box RNA helicase Prp43. eLife **6**, e21510 (2017). 28092261 10.7554/eLife.21510 PMC5262380

[9] F. Hamann, M. Enders, R. Ficner, Structural basis for RNA translocation by DEAH-box ATPases. Nucleic Acids Res. **47**, 4349–4362 (2019). 30828714 10.1093/nar/gkz150 PMC6486627

[10] K. E. Bohnsack, R. Ficner, M. T. Bohnsack, S. Jonas, Regulation of DEAH-box RNA helicases by G-patch proteins. Biol. Chem. **402**, 561–579 (2021). 33857358 10.1515/hsz-2020-0338

[11] F. De Bortoli, S. Espinosa, R. Zhao, DEAH-box RNA helicases in pre-mRNA splicing. Trends Biochem. Sci. **46**, 225–238 (2021). 33272784 10.1016/j.tibs.2020.10.006 PMC8112905

[12] P. Linder, E. Jankowsky, From unwinding to clamping—The DEAD box RNA helicase family. Nat. Rev. Mol. Cell Biol. **12**, 505–516 (2011). 21779027 10.1038/nrm3154

[13] I. Jarmoskaite, R. Russell, RNA helicase proteins as chaperones and remodelers. Annu. Rev. Biochem. **83**, 697–725 (2014). 24635478 10.1146/annurev-biochem-060713-035546 PMC4143424

[14] K. E. Bohnsack, N. Kanwal, M. T. Bohnsack, Prp43/DHX15 exemplify RNA helicase multifunctionality in the gene expression network. Nucleic Acids Res. **50**, 9012–9022 (2022). 35993807 10.1093/nar/gkac687 PMC9458436

[15] K. E. Sloan, M. T. Bohnsack, Unravelling the mechanisms of RNA helicase regulation. Trends Biochem. Sci. **43**, 237–250 (2018). 29486979 10.1016/j.tibs.2018.02.001

[16] M. E. Fairman , Protein displacement by DExH/D “RNA helicases” without duplex unwinding. Science **304**, 730–734 (2004). 15118161 10.1126/science.1095596

[17] E. Jankowsky, C. H. Gross, S. Shuman, A. M. Pyle, Active disruption of an RNA-protein interaction by a DExH/D RNA helicase. Science **291**, 121–125 (2001). 11141562 10.1126/science.291.5501.121

[18] L. Aravind, E. V. Koonin, G-patch: A new conserved domain in eukaryotic RNA-processing proteins and type D retroviral polyproteins. Trends Biochem. Sci. **24**, 342–344 (1999). 10470032 10.1016/s0968-0004(99)01437-1

[19] C. Bolinger, A. Sharma, D. Singh, L. Yu, K. Boris-Lawrie, RNA helicase A modulates translation of HIV-1 and infectivity of progeny virions. Nucleic Acids Res. **38**, 1686–1696 (2010). 20007598 10.1093/nar/gkp1075 PMC2836548

[20] M. K. Studer, L. Ivanovic, M. E. Weber, S. Marti, S. Jonas, Structural basis for DEAH-helicase activation by G-patch proteins. Proc. Natl. Acad. Sci. U.S.A. **117**, 7159–7170 (2020). 32179686 10.1073/pnas.1913880117 PMC7132122

[21] F. Hamann , Structural analysis of the intrinsically disordered splicing factor Spp2 and its binding to the DEAH-box ATPase Prp2. Proc. Natl. Acad. Sci. U.S.A. **117**, 2948–2956 (2020). 31974312 10.1073/pnas.1907960117 PMC7022188

[22] K.-T. Jeang, V. Yedavalli, Role of RNA helicases in HIV-1 replication. Nucleic Acids Res. **34**, 4198–4205 (2006). 16935887 10.1093/nar/gkl398 PMC1616970

[23] G. Singh, X. Heng, K. Boris-Lawrie, “Chapter 7—Cellular RNA helicases support early and late events in retroviral replication” in Retrovirus-Cell Interactions, L. J. Parent, Ed. (Academic Press, 2018), pp. 253–271, 10.1016/B978-0-12-811185-7.00007-8.

[24] S. M. Heaton, P. R. Gorry, N. A. Borg, DExD/H-box helicases in HIV-1 replication and their inhibition. Trends Microbiol. **31**, 393–404 (2023). 36463019 10.1016/j.tim.2022.11.001

[25] C.-Y. Chen, X. Liu, K. Boris-Lawrie, A. Sharma, K.-T. Jeang, Cellular RNA helicases and HIV-1: Insights from genome-wide, proteomic, and molecular studies. Virus Res. **171**, 357–365 (2013). 22814432 10.1016/j.virusres.2012.06.022 PMC3493675

[26] Z. Kohoutova , The impact of altered polyprotein ratios on the assembly and infectivity of Mason-Pfizer monkey virus. Virology **384**, 59–68 (2009). 19062065 10.1016/j.virol.2008.10.048 PMC3779691

[27] A. Zabransky , Three active forms of aspartic proteinase from Mason-Pfizer monkey virus. Virology **245**, 250–256 (1998). 9636364 10.1006/viro.1998.9173

[28] M. Rumlova-Klikova, E. Hunter, M. V. Nermut, I. Pichova, T. Ruml, Analysis of Mason-Pfizer monkey virus gag domains required for capsid assembly in bacteria: Role of the N-terminal proline residue of CA in directing particle shape. J. Virol. **74**, 8452–8459 (2000). 10954545 10.1128/jvi.74.18.8452-8459.2000 PMC116356

[29] O. Barabas , dUTPase and nucleocapsid polypeptides of the Mason-Pfizer monkey virus form a fusion protein in the virion with homotrimeric organization and low catalytic efficiency. J. Biol. Chem. **278**, 38803–38812 (2003). 12869552 10.1074/jbc.M306967200

[30] H. Bauerova-Zabranska , The RNA binding G-patch domain in retroviral protease is important for infectivity and D-type morphogenesis of Mason-Pfizer monkey virus. J. Biol. Chem. **280**, 42106–42112 (2005). 16257973 10.1074/jbc.M508031200

[31] I. Křížová , The G-patch domain of Mason-Pfizer monkey virus is a part of reverse transcriptase. J. Virol. **86**, 1988–1998 (2012). 22171253 10.1128/JVI.06638-11 PMC3302395

[32] B. B. Roy , Association of RNA helicase a with human immunodeficiency virus type 1 particles. J. Biol. Chem. **281**, 12625–12635 (2006). 16527808 10.1074/jbc.M510596200

[33] F. K. Schur , Structure of the immature HIV-1 capsid in intact virus particles at 8.8 A resolution. Nature **517**, 505–508 (2015). 25363765 10.1038/nature13838

[34] I. Memet, C. Doebele, K. E. Sloan, M. T. Bohnsack, The G-patch protein NF-kappaB-repressing factor mediates the recruitment of the exonuclease XRN2 and activation of the RNA helicase DHX15 in human ribosome biogenesis. Nucleic Acids Res. **45**, 5359–5374 (2017). 28115624 10.1093/nar/gkx013 PMC5435916

[35] Z. Niu, W. Jin, L. Zhang, X. Li, Tumor suppressor RBM5 directly interacts with the DExD/H-box protein DHX15 and stimulates its helicase activity. FEBS Lett. **586**, 977–983 (2012). 22569250 10.1016/j.febslet.2012.02.052

[36] R. Yoshimoto, N. Kataoka, K. Okawa, M. Ohno, Isolation and characterization of post-splicing lariat-intron complexes. Nucleic Acids Res. **37**, 891–902 (2009). 19103666 10.1093/nar/gkn1002 PMC2647322

[37] N. Tanaka, A. Aronova, B. Schwer, Ntr1 activates the Prp43 helicase to trigger release of lariat-intron from the spliceosome. Genes. Dev. **21**, 2312–2325 (2007). 17875666 10.1101/gad.1580507 PMC1973145

[38] S. Lebaron , The ATPase and helicase activities of Prp43p are stimulated by the G-patch protein Pfa1p during yeast ribosome biogenesis. EMBO J. **28**, 3808–3819 (2009). 19927118 10.1038/emboj.2009.335 PMC2797057

[39] Y. L. Chen , The telomerase inhibitor Gno1p/PINX1 activates the helicase Prp43p during ribosome biogenesis. Nucleic Acids Res. **42**, 7330–7345 (2014). 24823796 10.1093/nar/gku357 PMC4066782

[40] V. N. Uversky, Intrinsically disordered proteins and their environment: Effects of strong denaturants, temperature, pH, counter ions, membranes, binding partners, osmolytes, and macromolecular crowding. Protein J. **28**, 305–325 (2009). 19768526 10.1007/s10930-009-9201-4

[41] M. Castoralova , A myristoyl switch at the plasma membrane triggers cleavage and oligomerization of Mason-Pfizer monkey virus matrix protein. eLife **13**, e93489 (2024). 38517277 10.7554/eLife.93489 PMC11014724

[42] A. U. Heininger , Protein cofactor competition regulates the action of a multifunctional RNA helicase in different pathways. RNA Biol. **13**, 320–330 (2016). 26821976 10.1080/15476286.2016.1142038 PMC4829300

[43] M. A. Fouraux , The human La (SS-B) autoantigen interacts with DDX15/hPrp43, a putative DEAH-box RNA helicase. RNA **8**, 1428–1443 (2002). 12458796 10.1017/s1355838202021076 PMC1370349

[44] A. Dostalkova , Mutations in the basic region of the Mason-Pfizer monkey virus nucleocapsid protein affect reverse transcription, genomic RNA packaging, and the virus assembly site. J. Virol. **92**, e00106-18 (2018). 29491167 10.1128/JVI.00106-18 PMC5923082

[45] S. Rao, T. Mahmoudi, DEAD-ly affairs: The roles of DEAD-box proteins on HIV-1 viral RNA metabolism. Front. Cell Dev. Biol. **10**, 917599 (2022). 35769258 10.3389/fcell.2022.917599 PMC9234453

[46] B. B. Roy , Association of RNA helicase A with human immunodeficiency virus type 1 particles*. J. Biol. Chem. **281**, 12625–12635 (2006). 16527808 10.1074/jbc.M510596200

[47] A. Sharma, K. Boris-Lawrie, Determination of host RNA helicases activity in viral replication. Methods Enzymol. **511**, 405–435 (2012). 22713331 10.1016/B978-0-12-396546-2.00019-X PMC4862593

[48] T. G. Müller, V. Zila, B. Müller, H.-G. Kräusslich, Nuclear capsid uncoating and reverse transcription of HIV-1. Ann. Rev. Virol. **9**, 261–284 (2022). 35704745 10.1146/annurev-virology-020922-110929

[49] V. Veverka , Three-dimensional structure of a monomeric form of a retroviral protease. J. Mol. Biol. **333**, 771–780 (2003). 14568536 10.1016/j.jmb.2003.08.049

[50] H. M. Hanson, N. A. Willkomm, H. Yang, L. M. Mansky, Human retrovirus genomic RNA packaging. Viruses **14**, 1094 (2022). 35632835 10.3390/v14051094 PMC9142903

[51] G. M. Pocock, J. T. Becker, C. M. Swanson, P. Ahlquist, N. M. Sherer, HIV-1 and M-PMV RNA nuclear export elements program viral genomes for distinct cytoplasmic trafficking behaviors. PLoS Pathog. **12**, e1005565 (2016). 27070420 10.1371/journal.ppat.1005565 PMC4829213

[52] J. K. Maldonado Rebecca , Visualizing association of the retroviral gag protein with unspliced viral RNA in the nucleus. mBio **11**, e00524-20 (2020), 10.1128/mbio.00524-00520. 32265329 PMC7157774

[53] L. Z. Scheifele, R. A. Garbitt, J. D. Rhoads, L. J. Parent, Nuclear entry and CRM1-dependent nuclear export of the Rous sarcoma virus Gag polyprotein. Proc. Natl. Acad. Sci. U.S.A. **99**, 3944–3949 (2002). 11891341 10.1073/pnas.062652199 PMC122628

[54] R. Garbitt-Hirst, P. Kenney Scott, J. Parent Leslie, Genetic evidence for a connection between Rous Sarcoma Virus Gag nuclear trafficking and genomic RNA packaging. J. Virol. **83**, 6790–6797 (2009). 19369339 10.1128/JVI.00101-09 PMC2698546

[55] J. N. Sfakianos, R. A. LaCasse, E. Hunter, The M-PMV cytoplasmic targeting-retention signal directs nascent Gag polypeptides to a pericentriolar region of the cell. Traffic **4**, 660–670 (2003). 12956869 10.1034/j.1600-0854.2003.00125.x

[56] A. E. Pasquinelli , The constitutive transport element (CTE) of Mason-Pfizer monkey virus (MPMV) accesses a cellular mRNA export pathway. EMBO J. **16**, 7500–7510 (1997). 9405378 10.1093/emboj/16.24.7500 PMC1170349

[57] Y. Li , An intron with a constitutive transport element is retained in a Tap messenger RNA. Nature **443**, 234–237 (2006). 16971948 10.1038/nature05107

[58] C. R. Edwards , A dynamic intron retention program in the mammalian megakaryocyte and erythrocyte lineages. Blood **127**, e24–e34 (2016). 26962124 10.1182/blood-2016-01-692764 PMC4850870

[59] H. Dvinge, R. K. Bradley, Widespread intron retention diversifies most cancer transcriptomes. Genome Med. **7**, 45 (2015). 26113877 10.1186/s13073-015-0168-9 PMC4480902

[60] B. Müller , Construction and characterization of a fluorescently labeled infectious human immunodeficiency virus type 1 derivative. J. Virol. **78**, 10803–10813 (2004). 15367647 10.1128/JVI.78.19.10803-10813.2004 PMC516407

[61] M. Obr , Stabilization of the beta-hairpin in Mason-Pfizer monkey virus capsid protein—A critical step for infectivity. Retrovirology **11**, 94 (2014). 25365920 10.1186/s12977-014-0094-8 PMC4219007

[62] M. Rumlova , Breast cancer-associated protein—A novel binding partner of Mason-Pfizer monkey virus protease. J. Gen. Virol. **95**, 1383–1389 (2014). 24659101 10.1099/vir.0.064345-0

[63] R. Hadravova , In vitro assembly of virus-like particles of a gammaretrovirus, the murine leukemia virus XMRV. J. Virol. **86**, 1297–1306 (2012). 22090120 10.1128/JVI.05564-11 PMC3264384

[64] R. Hadravova, M. Rumlova, T. Ruml, FAITH—Fast assembly inhibitor test for HIV. Virology **486**, 78–87 (2015). 26410239 10.1016/j.virol.2015.08.029

[65] W. F. Vranken , The CCPN data model for NMR spectroscopy: Development of a software pipeline. Proteins **59**, 687–696 (2005). 15815974 10.1002/prot.20449

[66] M. Rumlova, I. Krizova, J. Zelenka, J. Weber, T. Ruml, Does BCA3 play a role in the HIV-1 replication cycle? Viruses **10**, 212 (2018). 29677171 10.3390/v10040212 PMC5923506

[67] W. E. Diehl, E. Stansell, S. M. Kaiser, M. Emerman, E. Hunter, Identification of postentry restrictions to Mason-Pfizer monkey virus infection in New World monkey cells. J. Virol. **82**, 11140–11151 (2008). 18799582 10.1128/JVI.00269-08 PMC2573280

[68] G. Z. Wang, S. P. Goff, Postentry restriction of Mason-Pfizer monkey virus in mouse cells. J. Virol. **89**, 2813–2819 (2015). 25540373 10.1128/JVI.03051-14 PMC4325713

[69] S. B. Kutluay, P. D. Bieniasz, Analysis of HIV-1 Gag-RNA Interactions in cells and virions by CLIP-seq. Methods Mol. Biol. **1354**, 119–131 (2016). 26714708 10.1007/978-1-4939-3046-3_8 PMC6548315

[70] P. Danecek , Twelve years of SAMtools and BCFtools. GigaScience **10**, giab008 (2021). 33590861 10.1093/gigascience/giab008 PMC7931819

[71] P. Junková, M. Rumlová, Data from “Proteomic analysis of Mason-Pfizer monkey virus virions, project accession: PXD046672”. PRIDE. http://www.ebi.ac.uk/pride/archive/projects/PXD046672. Deposited 5 November 2023.

