# Supplementary material for: Unveiling the DHX15–G-patch interplay in retroviral RNA packaging
Source: Proc Natl Acad Sci U S A. 2024 Sep 25;121(40):e2407990121. doi: 10.1073/pnas.2407990121 (PMC11459146; doi:10.1073/pnas.2407990121)
Supplement: Supplementary file 1 — Appendix 01 (PDF) [file pnas.2407990121.sapp.pdf]

PNAS

Supporting Information for
Unveiling the DHX15 - G-patch Interplay in Retroviral RNA Packaging

Alžběta Dostálková[1], Ivana Křížová[1], Petra Junková2[2,3], Jana Racková[1], Marina Kapisheva[1], Radim Novotný[3], Matěj Danda[1], Karolína Zvonařová[1], Larisa Šinkovec[1], Kateřina Večerková[4,5], Lucie Bednářová[2], Tomáš Ruml[3] and Michaela Rumlová[1]*

[1]Department of Biotechnology, University of Chemistry and Technology, Prague, 166 28 Prague, Czech Republic
[2]Institute of Organic Chemistry and Biochemistry IOCB Research Centre & Gilead Sciences, Czech Academy of Sciences, Flemingovo nám. 2, 166 10, Prague, Czech Republic
[3]Department of Biochemistry and Microbiology, University of Chemistry and Technology, Prague, 166 28 Prague, Czech Republic
[4]Department of Informatics and Chemistry, University of Chemistry and Technology, Prague, 166 28 Prague, Czech Republic
[5]Institute of Molecular Genetics, Czech Academy of Sciences, Vídeňská 1083, 142 20, Prague, Czech Republic

*Corresponding author: Michaela Rumlová
michaela.rumlova@vscht.cz

This PDF file includes:

Supporting text
Figures S1 to S2
Tables S1
Legends for Datasets S1
SI References

Other supporting materials for this manuscript include the following:

Datasets S1

Supporting Information Text

Material and Methods

Isolation and purification of M-PMV virions for mass spectrometry analysis

At 72 h post-transfection of HEK 293 cells with the M-PMV proviral vector pSARM4, culture medium from 22 Petri dishes (100 mm diameter) was collected, centrifuged for 5 min at 1,500 rpm and filtered through a 0.45 µm filter. The sample was then centrifuged through a 20% sucrose cushion, and the viral pellet was gently resuspended in PBS. It was then purified through an iodixanol (OptiprepTM) density gradient as previously described (1). The fractions containing virions, corresponding to an OptiprepTM concentration from 12 to 18%, were combined, diluted with PBS and pelleted by ultracentrifugation for 90 min at 39,000 rpm and 4 °C in a SW41Ti rotor. The viral pellets were resuspended in a final volume of 100 µl of PLB 2x and analyzed using mass spectrometry.

Preparation of samples for mass spectrometry analysis

Mass spectrometry analysis was performed with the samples of wild-type and ΔGP M-PMV virions, both in three biological replicates (obtained from three independent isolations of virions). Additionally, mock samples were also analyzed in three biological replicates.

Isolated and purified M-PMV virions were separated by the short-line SDS-PAGE using 15% separation and 5% resolving gels. 5 µg of total protein were loaded into the line of each sample and the samples of virions were analyzed in three technical repetitions. The SDS-PAGE was performed at 180 V for 20 min to separate the virion samples in a separation gel of approximately 1.5 cm long distance. Proteins were then stained by Imperial™ Protein Stain (Thermo Scientific) and whole parts of lines containing the separated virions were cut into small pieces followed by in-gel digestion of proteins. Briefly, gel pieces were initially destained by the solution of 0.1M ammonium bicarbonate buffer (ABC) mixed with acetonitrile (ACN) in ratio of 1:1 (v/v). Gel pieces were then dried by ACN, reduced by 10mM dithiotreitol dissolved in 0.1M ABC at 56 °C for 45 min and alkylated by 55mM iodoacetamide dissolved in 0.1M ABC at room temperature for 30 min in dark. Gel pieces were then washed in 0.1M ABC:ACN 1:1 (v/v) and dried by ACN. 35 µl of the 12.5 ng.µl^{-1} solution of Sequencing Grade Trypsin (Sigma) dissolved in 50mM ABC were add to the the dries gel pieces and incubated for 30 min at 4 °C. Gel pieces were then overlaid by 35 µl of 50 mM ABC and incubated overnight in 37 °C. Peptides were from the gel pieces extracted by the three subsequent sonications in 35% ACN/0.1% trifluoroacetic acid (TFA) solution (sonicated twice) and in 70% ACN/0.1% TFA solution (sonicated once). After each sonication the solution was removed and changed to the new one. Each removed solutions were combined into one final sample, lyophilized, resuspended in 20 µl of 0.1% TFA, and desalted by Pierce™ C18 Tips (Thermo Scientific) according to the manufacturer's instructions.

Mass spectrometry analysis

Mass spectrometry analysis was performed with the samples of wild-type and ΔGP M-PMV virions, both in three biological replicates (obtained from three independent isolations of virions). Additionally, mock samples were also analysed in three biological replicates.

Measurements were carried out using a UHPLC Dionex Ultimate3000 RSLC nano (Dionex) connected to an ESI-Q/TOF Maxis Impact mass spectrometer (Bruker Daltonics). Lyophilized samples dissolved in a 97:3:0.1 % mixture of water:acetonitrile:formic acid were loaded into an Acclaim PepMap 100 C18 trap column (100 µm x 2 cm, size of reverse phase particles 5 µm; Dionex) with a flow rate during the mobile phase of A 5 µl/min for 5 minutes. The peptides were then eluted from the trap column into an Acclaim PepMap RSLC C18 analytical column (75 µm x 150 mm, size of reverse phase particles 2 µm; Dionex) using the following gradient: 0 min 3 % B, 5 min 3 % B, 95 min 35 % B, 97 min 90 % B, 110 min 90 % B, 112 min 3 % B, 120 min 3 % B. The mobile phase A consisted of 0.1% formic acid in water and mobile phase B of 0.1% formic acid in acetonitrile. The flow rate during gradient separation was set at 0.3 µl/min. The peptides were eluted directly to an ESI source – Captive spray (Bruker Daltonics). Measurements were carried out in DDA positive ion mode with a precursor selection in the range of 400–1400 Da; up to ten precursors were selected for fragmentation from each MS spectrum.

Label-free protein quantification

Identification and LFQ quantification of proteins were performed in the MaxQuant software (version 1.6.3.4), using Andromeda search engine (http://www.coxdocs.org/doku.php). Database of human proteins and proteins of M-PMV downloaded from Uniprot database (September 2020) were used

for the protein identification. Database search parameters were set as follows: oxidation of methionine and acetylation of protein N-terminus as variable modification, carbamidomethylation of cysteins as fixed modification, mass tolerance in MS mode of 0.07 Da in the first search and 0.006 Da in the main search; MS/MS mode of 40 ppm; enzyme trypsin, two missed cleavage sites allowed per peptide; minimal peptide length of 7 residues, maximal peptide mass of 4.600 Da. Proteins and peptides were identified with FDR 0.01. Quantification was performed in LFQ mode with LFQ min. ratio count of 2 and both, unique and razor, peptides were used for quantification.
MaxQuant output files were further statistically processed in the Perseus software (version 1.6.5.0). Proteins identified only by site, matching to the reverse database, marked as potential contaminants or identified in mock samples were excluded from the analysis. For remaining proteins: LFQ intensity values were transformed to a logarithmic scale using log2(x); missing data were replaced by the values of the noise from a normal distribution. To reveal changes in protein abundances among wild-type and ΔGPD M-PMV virions, t-test was used. FDR was set to 0.01, s0 to 0.1 and number of permutations to 250. To each protein the difference value (representing difference in protein distribution) and -log(p-value) (representing statistical significance) was assigned and the proteins with significantly lowered abundance in ΔGPD M-PMV virions compared to those in wild-type were labeled. Data were exported and further proceeded in MS Excel. The mass spectrometry proteomics data have been deposited to the ProteomeXchange Consortium via the PRIDE (2) partner repository with the dataset identifier PXD046672.

Protein production and purification

GST-TEV-GP was produced in *E. coli* BL21(DE3). The cells were grown at 37 °C in LB media until they reached an OD_{600} of 0.8, at which point protein expression was induced by IPTG (final concentration 0.4 mM). At 4 h post-induction, the cells were harvested. The cell pellet was resuspended in lysis buffer (50 mM HEPES, pH 7.5, 200 mM NaCl, 2 mM mercaptoethanol, 2 mM $MgCl_2$, 10% glycerol) and lysed using a standard One-shot procedure. After centrifugation at 50,000 × g for 20 min, the supernatant was applied onto a GST Trap column (Cytiva, USA), and the bound proteins were eluted with a glutathione gradient (2- to 20 mM glutathione over 30 min). The GST tag was removed using GST TEV protease added to the purified GST-TEV-GP protein during dialyses into lysis buffer. The resulting mixture of GP, GST tag and GST TEV protease was reapplied onto the GST Trap column. GP, which passed through the column, was concentrated and loaded onto a size-exclusion chromatography column (HiLoad 26/600 Superdex, Cytiva, USA), equilibrated in SEC buffer (50 mM HEPES, pH 7.5, 150 mM NaCl, 10% glycerol, 2 mM $MgCl_2$). Fractions containing M-PMV GP were combined, concentrated and stored in SEC buffer at -80°C.
His-3C-DHX15 (hereafter denoted DHX15) was produced in Sf9 cells for 96 h at 110 rpm and 27 °C. The cells were then harvested, and the cell pellet was resuspended in lysis buffer (50 mM HEPES, pH 7.5, 500 mM NaCl, 2 mM mercaptoethanol, 10% glycerol, 2 mM $MgCl_2$, 20 mM imidazole) and lysed using a standard One-shot procedure. Following centrifugation at 50,000 × g for 20 min, the supernatant was applied onto a Ni-NTA column (His TRAP HP column, Cytiva, USA). The bound proteins were eluted with 600 mM imidazole. Fractions containing DHX15 were dialyzed into Heparin loading buffer (50 mM HEPES, pH 7.5, 100 mM NaCl, 2 mM mercaptoethanol, 10% glycerol, 2 mM $MgCl_2$) and then loaded onto a heparin column (HiTRAP heparin HP column, Cytiva, USA). Elution was carried out using a 0.1 to 1 M NaCl gradient. Fractions containing DHX15 were dialyzed into SEC buffer (10 mM HEPES, pH 7.5, 200 mM NaCl, 2 mM DTT, 5% glycerol) and applied onto a size-exclusion chromatography column (HiLoad 26/600 Superdex, Cytiva, USA) equilibrated in SEC buffer. The collected fractions containing DHX15 were concentrated, aliquoted and stored in DHX15 storage buffer (50 mM HEPES, pH 7.5, 200 mM NaCl, 2 mM ME, 10% glycerol, 2 mM $MgCl_2$) at -80°C.

Circular dichroism (CD)

CD spectra were measured on a Jasco 1500 spectropolarimeter equipped with a Peltier thermostated holder PTC-517, as described previously (3, 4). ECD spectra in the far-UV spectral region (195 nm-280 nm) were obtained over a temperature range from 10 °C to 90 °C with 10 °C steps. The sample concentration was 0.11 mg/ml in a quartz cell with 0.5 mm path length. The following experimental setup was used: a scanning speed of 10 nm/min, a response time of 8 seconds, a scanning step of 0.1 nm, and standard instrument sensitivity. After baseline correction, spectra were expressed in terms of molar ellipticity (Δq)(deg.cm2.dmol-1) per residue. Numerical analysis of secondary structure was performed with the CDPro software package(5, 6).

siRNA knock-down
A set of siRNAs against DHX15 (3 unique 27mer siRNA duplexes A-UUA CUA AUA GCU GUC ACC AAA AGG GAC, B-GAA UUC AGU ACU GUG AAU AUU CCU UGG, C-UAU AUA GAA CUA CUU UCA AUA AAC UGC) was purchased from ORIGENE and their impact on DHX15 expression was evaluated by RT-qPCR and immunoanalysis. To analyze the impact of DHX15 knock-down on M-PMV, HEK 293 cells were seeded in a 12-well plate at a density of 3 x 10^5 cells/ml, and 24 h later, they were transfected with an equimolar mixture of all siRNAs at final concentrations of 5 nM and 10 nM using siTran 2.0 siRNA transfection reagent (ORIGENE). A second round of siRNA transfection was performed 24 h later, followed by transfection with the M-PMV proviral vector at 0.4 µg per well using X-tremeGENE HP (Roche). The next day, viral levels in the culture media were normalized by ELISA, and media were used to infect freshly seeded HEK 293 cells. Half of these infected cells was designated for RNA isolation and RT-qPCR analysis, while the remaining half was used to determine M-PMV infectivity.

Analysis of *dhx15* gene expression levels using RT-qPCR
HEK 293 cells were washed with PBS before total RNA was isolated using the RNeasy Mini Kit (Qiagen). A 5-µl aliquot of isolated RNA was used a template for reverse transcription with the RevertAid First Strand cDNA Synthesis Kit. To normalize and control both reverse transcription and qPCR reactions, TATAA universal RNA spike I (TATAA Biocenter) was added prior to reverse transcription. Expression of *dhx15* was determined by detecting exons 2, 4, and 13, using the following primers: Ex2F (5´-AGC TCA CTC AAC CCA CTC AG - 3´), Ex2R (5´- AAA CAG GGA GCT GAA GAC GTT -3´), Ex4F (CTGATGGGATGTTACTTCGTGA), Ex4R (5´- ATC TGT AGC CAG TGT CCT CTC -3´), Ex13F (5´- GCA CAT TTA GAA CGA ACA GGG CAT -3´), and Ex13R (5´- AGC ACC CAT TCA GGT TTG TGG -3´). Two endogenous genes, phospholipase A (PLA) and glyceraldehyde-3-phophate (GAPDH), were employed as reference genes. The following primers were used: PLAs (5´-AAG TTC TTG ATC CCC AAT GCT T-3') and PLAas (5´- GTC TGA TAG GAT GTG TTG GTT GC -3'); GAPDHs (5´-GAA GGT GAA GGT GGG AGT C -3') and GAPDHas (5´- GAA GAT GGT GAT GGG ATT TC -5'). qPCR reactions were performed in 96-well plates using a QuantStudio™ 5 Real-Time PCR System (Applied Biosystems™) under the following reaction conditions: 15 min at 95 °C, followed by 40 cycles of 30 s at 95 °C, 1 min at 60 °C, and 2 min at 72 °C. The level of gene expression was calculated using the ΔΔCq method.

Determination of RT activity after immunoprecipitation through GP and DHX15
At 48 h post-transfection of HEK 293 cells with wt and ΔGP pSARM4, medium containing M-PMV was filtered through 0.45 µm pores, and virions were concentrated by ultracentrifugation through a 20% sucrose cushion. The resulting pellet was resuspended in RT lysis buffer (50 mM Tris, pH 7.8, 80 mM KCl, 2.5 mM DTT, 0.75 mM EDTA, 0.5% Triton X-100, Halt™ Protease Inhibitor Cocktail) by gentle agitation for 2 h at 4 °C. Either Protein G magnetic beads conjugated with an antibody against DHX15 or Protein A magnetic beads conjugated with an antibody against M-PMV G-patch were added to the aliquoted viral suspensions. The mixtures were then incubated for 2 h at 4 °C. Magnetic beads with bound complex were washed with wash buffer (25 mM HEPES, 300 mM NaCl, 0.025% NP-40, 0.25 mM DTT, Halt™ Protease Inhibitor Cocktail) and resuspended in RT lysis buffer. Subsequently, RT activity was determined by RT-qPCR using artificial target SLA RNA (5´-AGU UGU UAG UCU ACG UGG ACC GAC AAA GAC AGA UUC UUU GAG GGA GCU AAG CUC AAC GUA GUU CUA ACA GUU UUU U-3´) where the immunoprecipitated complex was used as reverse transcriptase. The qPCR conditions were the same as described above. Reverse transcription products were calculated using the ΔΔCq method, and RT activity was determined as the relative percentage directly proportional to products and compared to the sample immunoprecipitated by antibody against M-PMV G-patch (considered 100%).

Generation of mScarlet-DHX15 producing HEK 293 cell line
One day prior to transfection, HEK 293 cells were seeded at a density of 3x10^5 cells/mL in a 24-well plate with 0.5 ml culture medium (DMEM +10%FBS) per well. For transfection, a mixture containing gRNA (7.5 pmol; sequence 5'-UAGGUCCAACCGGUGCCGCU-3'), EnGen® Spy Cas9 HF1 (7.5 pmol; NEB, USA) and dsDNA encoding mScarlet (0. 4 µg), prepared using 5'-Scarlet for 5'-CCTTAATAACTGCC CGTTCGAAGAGTGC GAGGATGGTGAGCA AGGGCGAGGCAGTG-3' and 3'-Scarlet rev 5'-AGAGGGGTAATCCT CCCCTAGGTCCAAC CGGTGCCGCTTGGA ACCGCTTCCCTTGT ACAGCTCGTCCATG CC-3' primers, based on the pWpXLd-Sc template. Lipofectamine CRISPRMAX Cas9 Reagent (1.5 µl; Invitrogen, USA) and Cas9 Plus Reagent (2.5

µl; Invitrogen, USA) were selected as transfection reagents. Five days after transfection, scarlet-positive HEK 293 cells were sorted into 96-well plates using the BD FACS Aria III single cell sorting method (excitation 569 nm, 586/15 bandpass emission filter). After 2-3 weeks, the grown HEK 293 colonies were analyzed by PCR to verify the insertion of the scarlet DHX15 gene and to distinguish between homozygous and heterozygous variants. Primers DHX15 for 5- TTTTAGCTGTTGGTT CCGGC-3 and Scarlet rev2 5- GTCCTCGAAGTTCA TCACGC-3 were used to verify gene insertion, while primers DHX15 for and DHX15 rev 5- GCCCAGAGAGAAAC AAAGGC-3 were used to distinguish homozygotes from heterozygotes. From selected clones, the gRNA (DNeasy Blood & Tissue Kits, QIAGEN, Germany) was isolated and sequenced to confirm specific genome editing. Sc-DHX15 protein expression was detected immunochemically using the MxDHX15 antibody (SCBT, USA) and by fluorescence microscopy.

Fluorecence Microscopy

mScarlet -DHX15 HEK 293 cells were grown on 35 mm dishes with glass bottom (MatTek) and transfected with wt MA-GFP M-PMV and ΔGP MA-GFP M-PMV proviral vectors, as described above. 24 hours post-transfection the cells were fixed in 4% paraformaldehyde in PBS at room temperature. Samples were then imaged with a spinning disc confocal microscope (Andor, Belfast, UK). For details and statistic see Supplementary information. The number of colocalization spots was analyzed in confocal series. For each infected cell, the number of colocalization spots was counted. A total of 58 cells transfected with wt MA-GFP M-PMV and 60 cells transfected with ΔGP MA-GFP M-PMV were analyzed. The difference in the number of colocalization spots per cell was assessed using the non-parametric Mann-Whitney U test. In cells transfected with wt MA-GFP M-PMV, an average of 2.05 colocalization spots (SD 0.88) were counted. In comparison, cells transfected with ΔGP MA-GFP M-PMV showed an average of 0.13 spots (SD 0.18). The difference was statistically significant with a p-value < 0.00001 (Mann-Whitney U test).

Photoactivatable-ribonucleoside-enhanced cross-linking and immunoprecipitation sequencing (PAR-CLIP-seq)

PAR-CLIP-seq experiments were based on a previously published protocol (7) with some modifications. HEK 293 cells cultured in 22 Petri dishes were transfected with pSARM4. As a negative control (mock), PEI was added into the medium of non-transfected cells. The next day, DMEM was replaced with DMEM containing 4-thiouridine to a final concentration of 100 µM. The cells were washed with ice-cold PBS and UV-crosslinked at an energy setting of 0.15 J/cm^2 in a UVP Crosslinker (Analytik Jena) with UV365nm bulbs. The cytosolic fraction was separated as described above, and Benzonase was added. After incubation at 37 °C for 20 min, each solution was divided into three aliquots and immunoprecipitated using Protein G magnetic beads conjugated with antibody against DHX15. Antibodies against M-PMV NC and M-PMV G-patch were used as controls for the immunoprecipitation. After a 2-h incubation at 4 °C, the samples were treated with Proteinase K at a final concentration 2 µg/µl for 30 min at 37 °C. RNA fragments were extracted according to the TRIzol extraction protocol. The cDNA library was prepared using NEXTFLEX® Small RNA-Seq Kit v3 according to the manufacturer's protocol. Barcoded samples were pooled and loaded onto cartridges included with MiSeq Reagent Kits v2. The library was sequenced using MiSeq platforms (Illumina, San Diego, CA, USA).

Bioinformatic analyses of PAR-CLIP-seq data to identify protein binding sites

Protein binding sites were identified using the nf-core/clipseq pipeline (8, 9) (version 1.0.0). The pipeline consists of adapter and quality trimming by Cutadapt (10) (version 3.0), genome mapping using STAR (11) (version 2.6.1d), crosslink identification by BEDTools (12) (version 2.29.2), and peak calling with multiple peak callers implemented. During the adapter and quality trimming step, the TGGAATTCTCGGGTGCCAAGG adapter for Illumina TrueSeq small RNA was removed from the 3' end of the reads, and reads shorter than 12 bp were filtered out as they mapped inconsistently. The trimmed reads were then mapped to the reference M-PMV genome (NCBI accession number NC_001550.1) using STAR aligner with arguments specifying that short reads are being aligned to a short genomic sequence (8,557 bp). Next, the peaks were deduced from the genome coverage and the frequency of T>C transitions characteristic to PAR-CLIP technology. Peaks were called using Piranha (13) (version 1.2.1) with the default bin size and cluster size of 3. Genome coverage was calculated using SAMtools (14) (version 1.14).

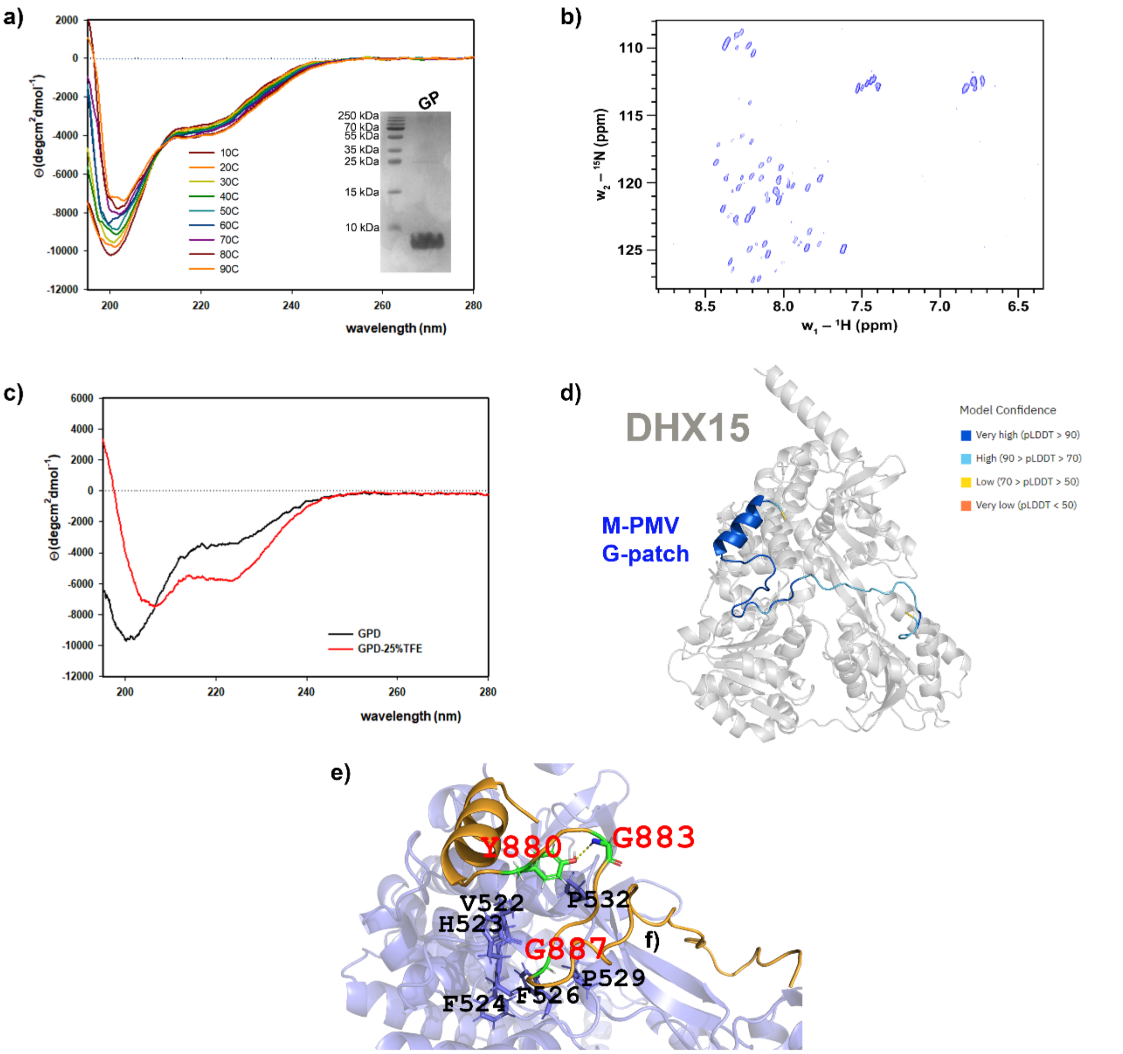

Fig. S1. Biochemical characterization of M-PMV G-patch peptide and validation of its binding to DHX15. (**a**) CD analysis of the temperature dependence of M-PMV G-patch peptide; inserted panel of Coomassie blue stained SDS PAGE of purified M-PMV G-patch peptide (aas 867-911). (**b**) 2D ^{1}H-^{15}N HSQC spectrum of uniformly ^{15}N-labeled M-PMV GP peptide at 298 K and pH 8. (**c**) CD spectra of M-PMV GP peptide in the absence (black) and presence (red) of TFE. (**d**) Confidence of Alphafold model of DHX15/M-PMV G-patch complex. (**e**) The detail of Alphafold model of DHX15/M-PMV G-patch complex: The aromatic residue Y880 forms a hydrogen bond with G883 and conserved G887 directs towards a DHX15 cavity consisting of V522, H523, F524 and F526 and P529.

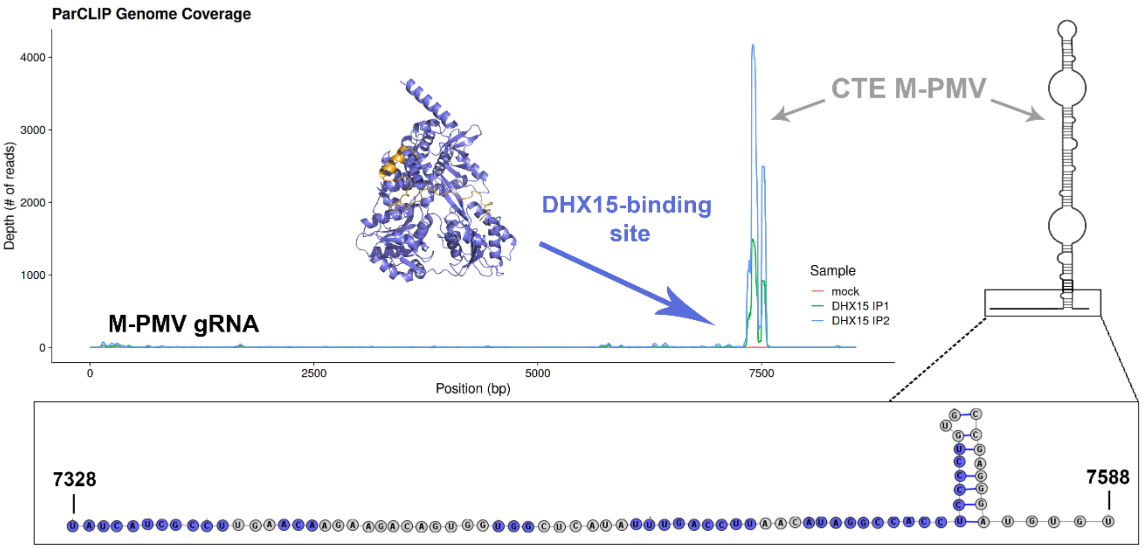

Fig. S2. **DHX15 binding sites from 7,328 -7,393 bp identified in M-PMV gRNA by PAR-CLIP.** HEK 293 cells producing M-PMV were incubated with 4-thiouridine and UV cross-linked. The cytosolic fraction of the infected cells was separated and used for immunoprecipitation with mouse antibody against DHX15 conjugated to magnetic beads. Following Proteinase K treatment, RNA was isolated from the immunoprecipitate and sequenced. DHX15 binding sites were identified by the nf-core/clipseq pipeline, as described in Materials & Methods. The number of reads based on the position in M-PMV gRNA from two biological experiments is shown. The sites of DHX15 binding on the M-PMV CTE region, whose structure is shown on the right, are labelled in blue. *P* values were assessed by analysis of variance with a combination of the Tukey-Kramer HSD test and the Scheffé, Bonferroni and Holm multiple comparison tests. *P* values (**, $P < 0.01$) (*, $P < 0.05$) were calculated by comparison to wt M-PMV, unless otherwise specified.

Table S1. HEK 293 cell proteins stably packed in wild-type M-PMV virions.

Protein ID [a]	Protein name	Gene name
P09543	2,3-cyclic-nucleotide 3-phosphodiesterase	CNP
P84077	ADP-ribosylation factor 1	ARF1
P40222	Alpha-taxilin	TXLNA
O95782	AP-2 complex subunit alpha-1	AP2A1
O94973	AP-2 complex subunit alpha-2	AP2A2
P63010	AP-2 complex subunit beta	AP2B1
O14965	Aurora kinase A	AURKA
P49674	Casein kinase I isoform epsilon	CSNK1E
P60033	CD81 antigen	CD81
Q53EZ4	Centrosomal protein of 55 kDa	CEP55
Q6P1N0	Coiled-coil and C2 domain-containing protein 1A	CC2D1A
P31689	DnaJ homolog subfamily A member 1	DNAJA1
P63167	Dynein light chain 1, cytoplasmic	DYNLL1
Q9H223	EH domain-containing protein 4	EHD4
P42566	Epidermal growth factor receptor substrate 15	EPS15
Q9UBC2	Epidermal growth factor receptor substrate 15-like 1	EPS15L1
Q0JRZ9	F-BAR domain only protein 2	FCHO2
Q9NUQ3	Gamma-taxilin	TXLNG
P49841	Glycogen synthase kinase-3 beta	GSK3B
Q9H444	Charged multivesicular body protein 4b	CHMP4B
Q15811	Intersectin-1	ITSN1
Q15366	Poly(rC)-binding protein 2	PCBP2
P11940	Polyadenylate-binding protein	PABPC1
O43143	Pre-mRNA-splicing factor ATP-dependent RNA helicase DHX15	DHX15
Q9P2B2	Prostaglandin F2 receptor negative regulator	PTGFRN
Q9UNF0	Protein kinase C and casein kinase substrate in neurons protein 2	PACSIN2
Q9UKS6	Protein kinase C and casein kinase substrate in neurons protein 3	PACSIN3
Q9HCE1	Putative helicase MOV-10	MOV10
P51153	Ras-related protein Rab-13	RAB13
P51149	Ras-related protein Rab-7a	RAB7A
P61224	Ras-related protein Rap-1b	RAP1B
Q7KZI7	Serine/threonine-protein kinase MARK2	MARK2
P53350	Serine/threonine-protein kinase PLK1	PLK1
Q99816	Tumor susceptibility gene 101 protein	TSG101
Q9UK41	Vacuolar protein sorting-associated protein 28 homolog	VPS28
Q9H9H4	Vacuolar protein sorting-associated protein 37B	VPS37B

O75351	Vacuolar protein sorting-associated protein 4B	VPS4B
Q9NP79	Vacuolar protein sorting-associated protein VTA1 homolog	VTA1

[a] UniProt reference sequence accession number.

<

Dataset S1 (separate file). Mass spectrometry (MS) analysis of cellular proteins incorporated into wild type (wt) and ∆GP-MPMV samples from three biological and three technical replicates.

SI References

1. F. K. Schur *et al.*, Structure of the immature HIV-1 capsid in intact virus particles at 8.8 A resolution. *Nature* **517**, 505-508 (2015).
2. Y. Perez-Riverol *et al.*, The PRIDE database resources in 2022: a hub for mass spectrometry-based proteomics evidences. *Nucleic Acids Res* **50**, D543-d552 (2022).
3. K. Strohalmova-Bohmova *et al.*, Role of Mason-Pfizer monkey virus CA-NC spacer peptide-like domain in assembly of immature particles. *J Virol* **88**, 14148-14160 (2014).
4. M. Dolezal *et al.*, Functional and Structural Characterization of Novel Type of Linker Connecting Capsid and Nucleocapsid Protein Domains in Murine Leukemia Virus. *J Biol Chem* **291**, 20630-20642 (2016).
5. N. Sreerama, R. W. Woody, On the analysis of membrane protein circular dichroism spectra. *Protein Sci* **13**, 100-112 (2004).
6. N. Sreerama, R. W. Woody, Estimation of protein secondary structure from circular dichroism spectra: comparison of CONTIN, SELCON, and CDSSTR methods with an expanded reference set. *Anal Biochem* **287**, 252-260 (2000).
7. S. B. Kutluay, P. D. Bieniasz, Analysis of HIV-1 Gag-RNA Interactions in Cells and Virions by CLIP-seq. *Methods Mol Biol* **1354**, 119-131 (2016).
8. P. Di Tommaso *et al.*, Nextflow enables reproducible computational workflows. *Nature Biotechnology* **35**, 316-319 (2017).
9. P. A. Ewels *et al.*, The nf-core framework for community-curated bioinformatics pipelines. *Nature Biotechnology* **38**, 276-278 (2020).
10. M. Martin, Cutadapt removes adapter sequences from high-throughput sequencing reads. *2011* **17**, 3 (2011).
11. A. Dobin *et al.*, STAR: ultrafast universal RNA-seq aligner. *Bioinformatics* **29**, 15-21 (2012).
12. A. R. Quinlan, I. M. Hall, BEDTools: a flexible suite of utilities for comparing genomic features. *Bioinformatics* **26**, 841-842 (2010).
13. P. J. Uren *et al.*, Site identification in high-throughput RNA–protein interaction data. *Bioinformatics* **28**, 3013-3020 (2012).
14. P. Danecek *et al.*, Twelve years of SAMtools and BCFtools. *GigaScience* **10** (2021).
